# Supplementary material for: Destabilization of NOXA mRNA as a common resistance mechanism to targeted therapies
Source: Nat Commun. 2019 Nov 14;10:5157. doi: 10.1038/s41467-019-12477-y (PMC6856172; doi:10.1038/s41467-019-12477-y)
Supplement: Supplementary file 1 — Supplementary Information [file 41467_2019_12477_MOESM1_ESM.pdf]

Destabilization of *NOXA* mRNA as a common resistance mechanism to targeted therapies

Montero *et al*.

Supplementary Information

Supplementary Figures

Supplementary Figure 1. (a,b) Knockdown efficiency of siRNA targeting anti-apoptotic family members. (a) mRNA in A375M (n=3) or (b) protein in SK-MEL-5 cells 72h after transfection of siRNAs. Statistical comparison relative to non-targeting control was done used t-test with correction using the Holm-Sidak method. *, adjusted P-value < 0.01. (c) Effect of siRNAs targeting *MCL1* on apoptosis induced by MEK inhibitor trametinib (100 nM) in A375M melanoma cells stably expressing EGFP or V5-tagged Mcl-1. (d) Effect of overexpressing Mcl-1 on apoptosis induced by BRAF inhibitor PLX4720 at indicated doses. (e) Dynamic BH3 profiling of A375M cells treated with Dabrafenib (1µM) for the indicated times. Dependence on anti-apoptotic proteins was evaluated with Bim peptide, whereas dependence on Mcl-1 was evaluated by treatment with NOXA peptide. Statistical comparison (n = 3-10) was done using ANOVA with Dunnett's multiple comparison test. *, adjusted P-value < 0.05; ***, adjusted P-value < 0.005; ****, adjusted P-value < 0.0001. (f) Dynamic BH3 profiling of A375M cells treated with dabrafenib, trametinib or both. Statistical comparison (n = 5) was done using one-way ANOVA with Dunnett's multiple comparison test. *, adjusted P-value < 0.05; **, adjusted P-value < 0.01; ***, adjusted P-value < 0.001; ****, adjusted P-value < 0.0001. (g) Dynamic BH3 profiling of *BRAF*-wild-type IPC298 cells after treatment with trametinib (n = 3-5). (h) Cell sorting of primary metastatic melanoma cells. Freshly isolated primary metastatic melanoma cells were sorted using BD FACSAria II. Cells were immunostained following the same protocol as for DBP: Live/Dead staining (Aqua), anti-CD45 (BV421) and anti-hNG2/MCSP (FITC). As shown, we were able to successfully sort CD45-MCSP+ (melanoma cells) from the rest of cells

found in the primary sample. Error bars indicate mean ± s.e.m of indicated replicates. Source data for all Western blots are provided as a Source Data file.

Supplemental Figure 1: Targeted therapies induce dependence on Mcl-1

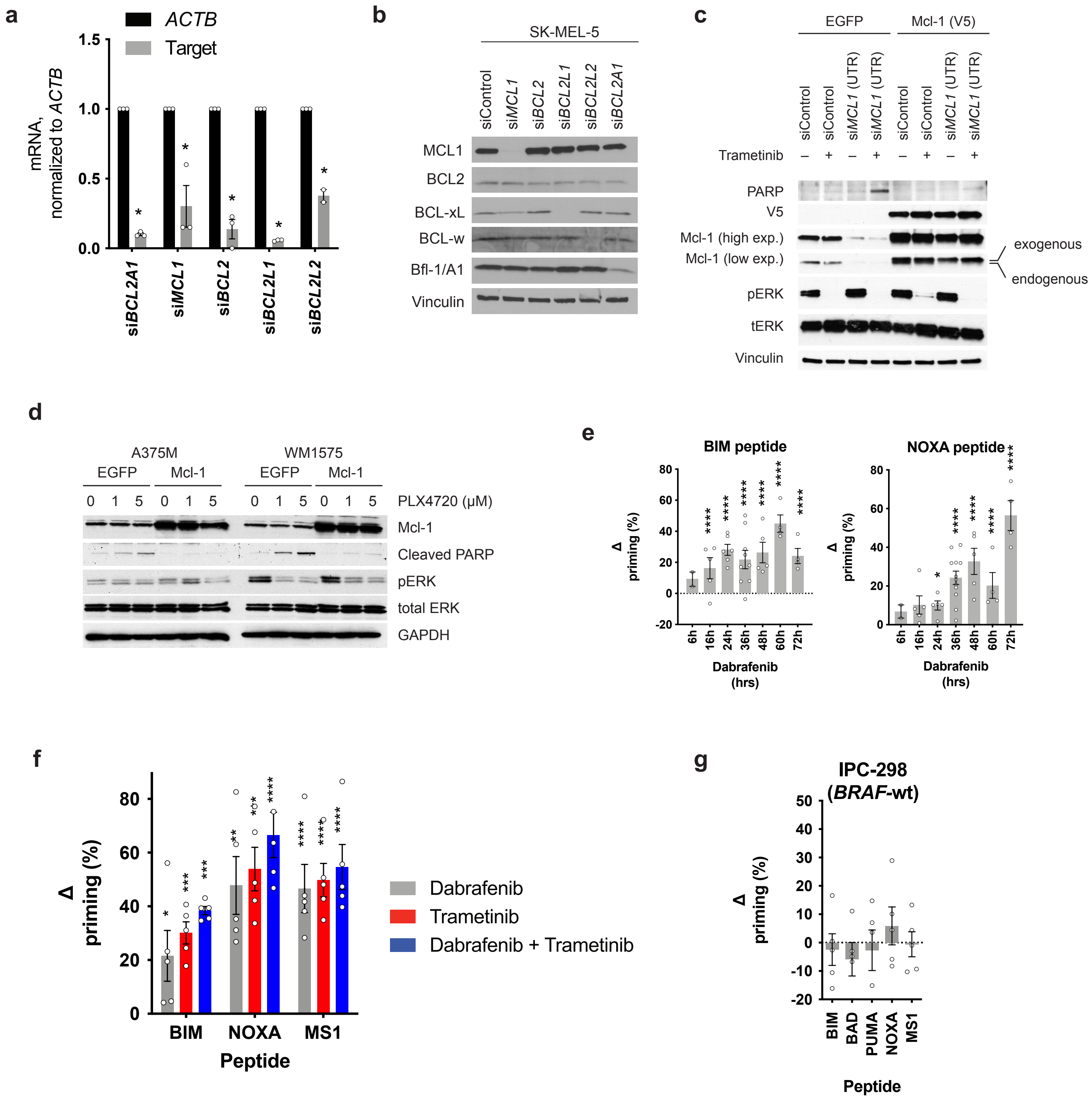

Supplemental Figure 1 (continued): Targeted therapies induce dependence on Mcl-1

h

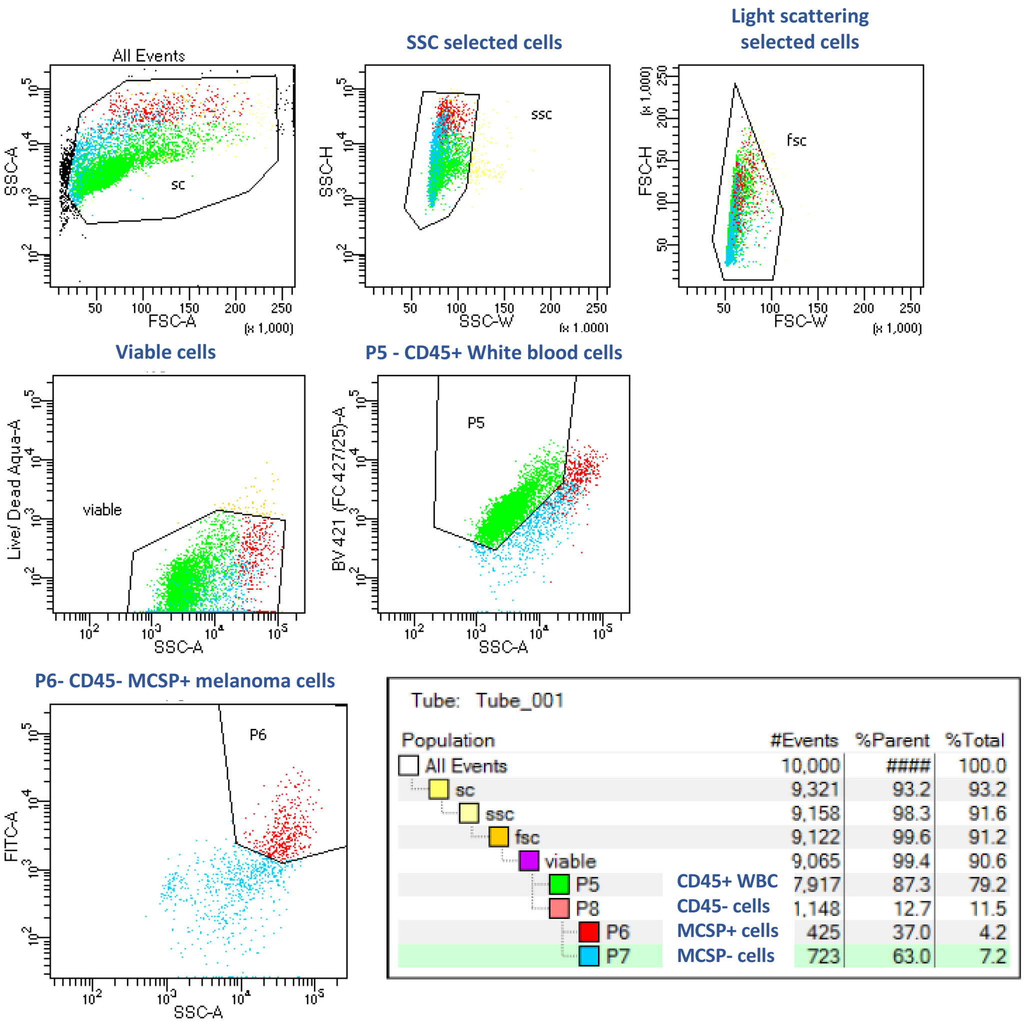

Tube: Tube_001

Population		#Events	%Parent	%Total
All Events		10,000	####	100.0
sc		9,321	93.2	93.2
ssc		9,158	98.3	91.6
fsc		9,122	99.6	91.2
viable		9,065	99.4	90.6
P5	CD45+ WBC	7,917	87.3	79.2
P8	CD45- cells	1,148	12.7	11.5
P6	MCSP+ cells	425	37.0	4.2
P7	MCSP- cells	723	63.0	7.2

Supplementary Figure 2. (a) Effect of BRAF and MEK inhibitors on NOXA protein in 3 *BRAF*-mutant melanoma cell lines. (b) Effect of MEK inhibitor on NOXA protein in *BRAF*-mutant colorectal cancer cell line. (c) Effect of imatinib on NOXA protein on *KIT*-mutant GIST882 cell line. (d) Effect of crizotinib on NOXA protein in *MET*-amplified cell lines. (e) Effect of crizotinib on NOXA protein in ALK-mutant cell lines. To detect phospho-ALK and total ALK in SH-SY5Y cells, total ALK was immunoprecipitated followed by Western with either total ALK or phospho-ALK antibodies. (f) Effect of lapatinib on NOXA protein in HER2-amplified breast cancer cell line. (g) Effect of gefitinib on NOXA protein in EGFR-mutant cell lung cancer cell line. (h) Effect of inhibitors of BRAF, MEK and PI3K on NOXA protein levels in A375M melanoma cell line. (i) Changes in NOXA mRNA following MEK inhibitor treatment in melanoma cell lines, as assessed in published microarray analysis (see Methods). For each gene, the expression is normalized to that of DMSO-treated cells. (j) Effect of inhibitors of oncogenic pathways on *NOXA* mRNA in published microarray analysis. (k) Effect of BRAF and MEK inhibitors on *NOXA* mRNA in primary melanocytes (n = 3). Statistical comparison to DMSO treated cells was done using one-way ANOVA with Dunnett's multiple comparison test. ****, $P < 0.0001$. (l,m) Effect of suppression of *ERK1* and *ERK2* by siRNA on Noxa protein (l) and (m) mRNA (n = 3). (n) Effect of concomitant suppression of BH3 proteins followed by siRNA targeting *MCL1* on cell growth. Statistical comparison was done using t-test with correction for multiple comparison using the Holm-Sidak method. **, adjusted P-value < 0.01; *, adjusted P-value < 0.05. Error bars indicate mean ± s.e.m of indicated replicates. Source data for all Western blots are provided as a Source Data file.

Supplementary Figure 2: Targeted therapies suppress *NOXA* mRNA

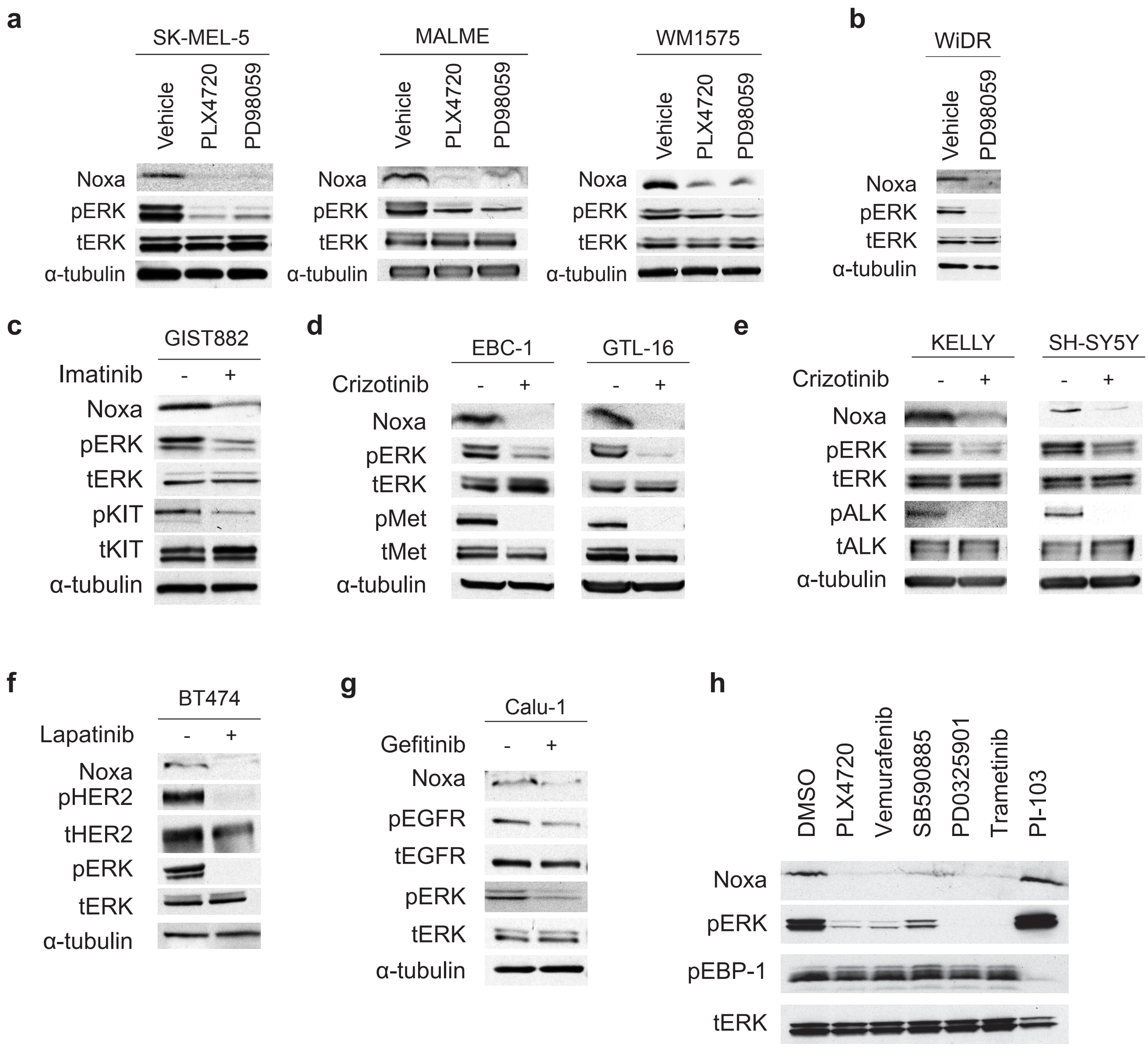

Supplementary Figure 2 (Part 2): Targeted therapies suppress *NOXA* mRNA

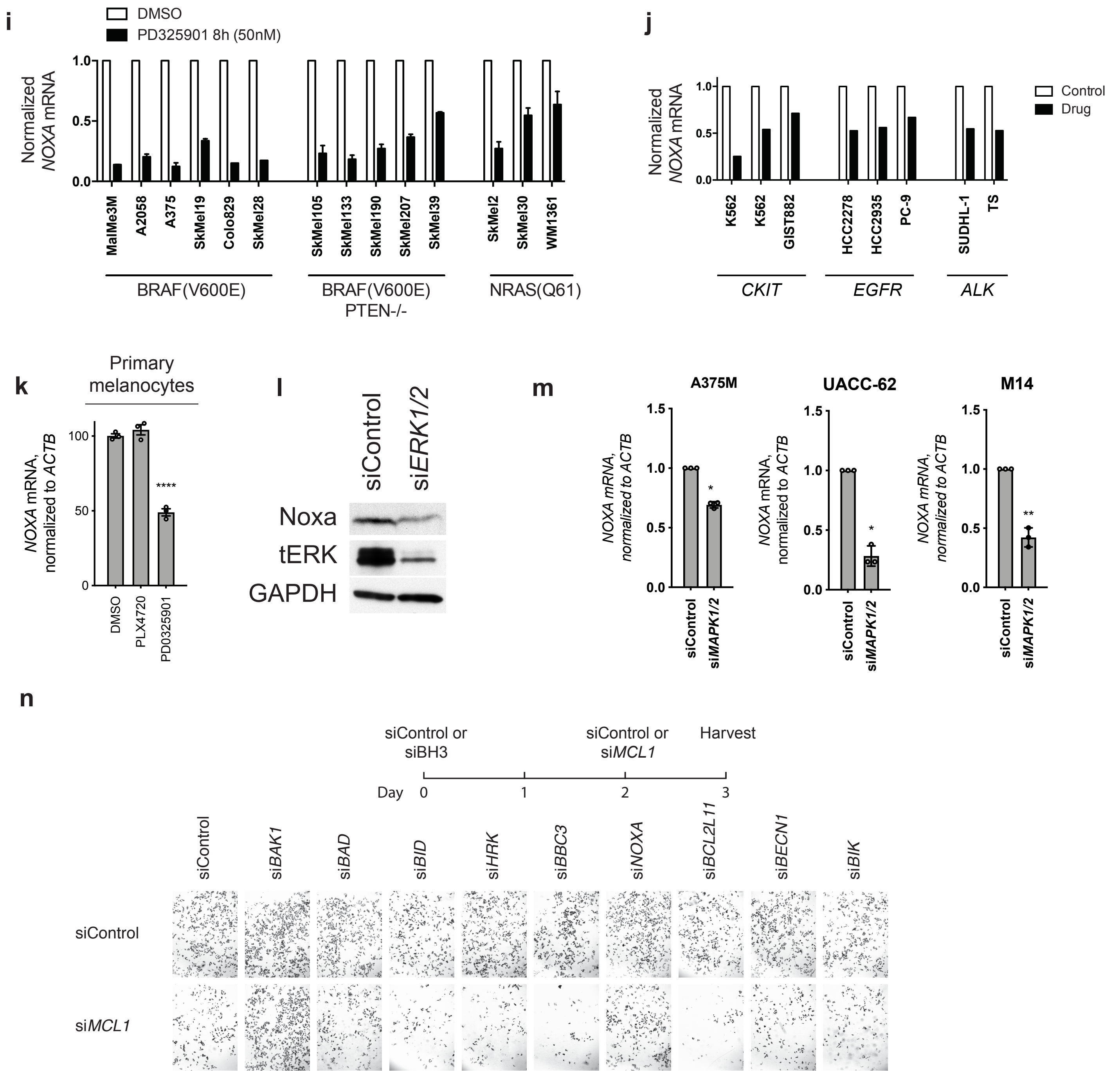

Supplementary Figure 3. The decay of *TRPM1* (a) and *HIF1A* (b) mRNA following treatment with actinomycin D with or without BRAF inhibitor treatment. Statistical comparison (n = 1-3) was done using extra-sum of squares F-test. ***, P < 0.001. (c) Effect of independent siRNAs targeting *ZFP36* on *NOXA* mRNA. Statistical comparison was done using ANOVA with Dunnett's correction for multiple comparisons. (d) Effect of *ZFP36* knockdown on the suppression of ERK signaling by dabrafenib (1µM). (e, f) Effect of ZFP36 expression on NOXA protein (e) and mRNA (f) upon treatment of GIST-T1 cells with imatinib. Statistical comparison was done using ANOVA with multiple comparisons using the Dunnett's test. ****, P < 0.001. (g,h) Effect of ZFP36 expression on *NOXA* mRNA in WM1575 cells or GTL-16 cells (h) (n = 3 each). Statistical comparison was done using unpaired t-test of vector compared to ZFP36 overexpressing cells, with multiple comparison correction using the Holm-Sidak method. ***, P < 0.01 (i) Immunoprecipitation of A375M cells expression either V5-tagged ZFP36 or control vector. Expression of *NOXA* mRNA following expression of ZFP36. Error bars indicate mean ± s.e.m of indicated replicates. Source data for all Western blots are provided as a Source Data file.

Supplemental Figure 3: ERK suppression decreases *NOXA* expression via TTP/ZFP36

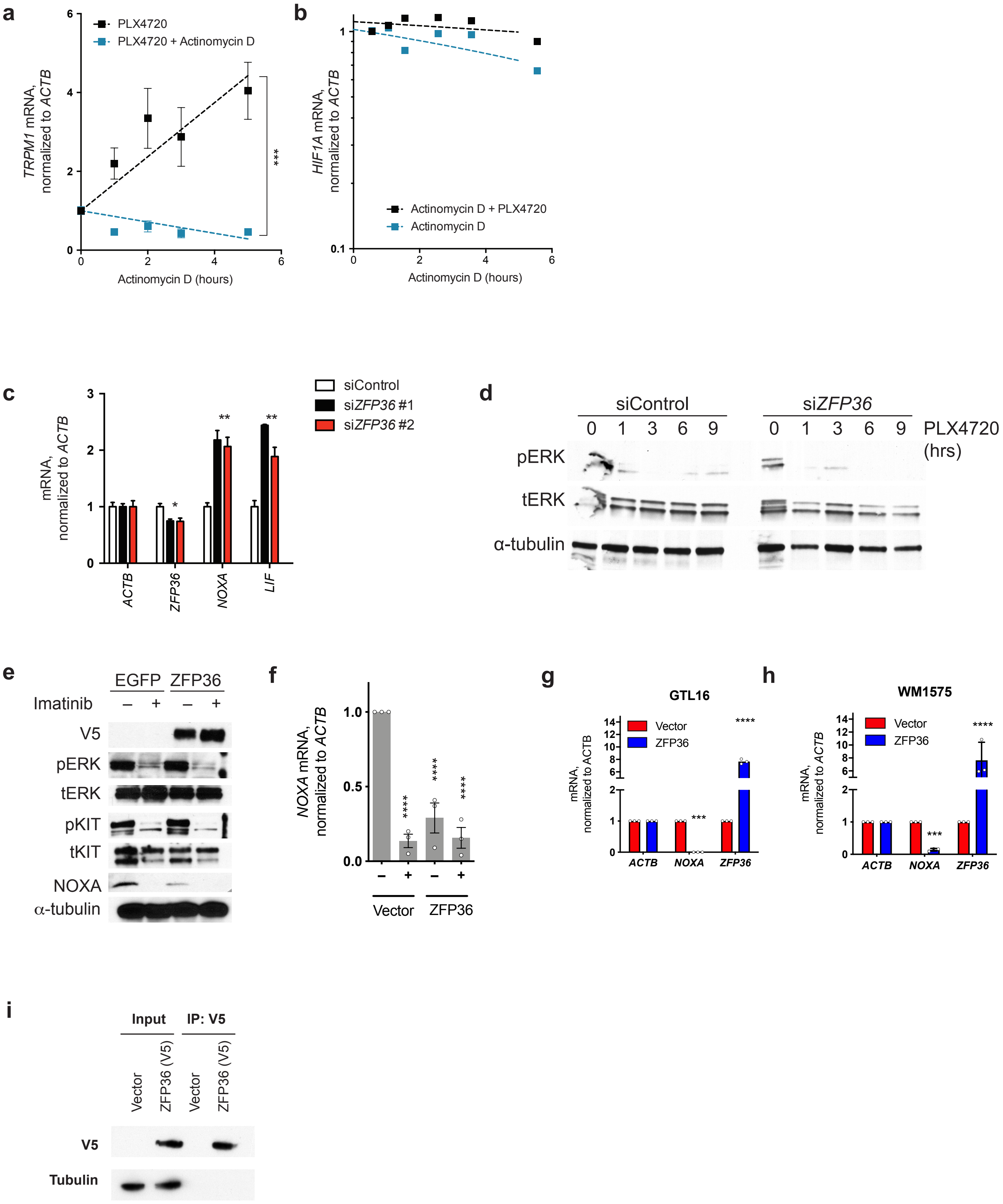

Supplementary Figure 4. (a) Effect of imatinib on total levels of NOXA and Bcl-2 family members in GIST-882 cells. (b) Association of Bim with Mcl-1 following imatinib treatment. (c) Association of Mcl-1 with Noxa and Bim following imatinib treatment. (d) Effect of A-1210477, dabrafenib or both on the association of Bim with Mcl-1 in A375 cells. (e) Effect of A1210477, dabrafenib or both in different sequential approaches on NOXA and BIM levels. Source data for all Western blots are provided as a Source Data file.

Supplemental Figure 4

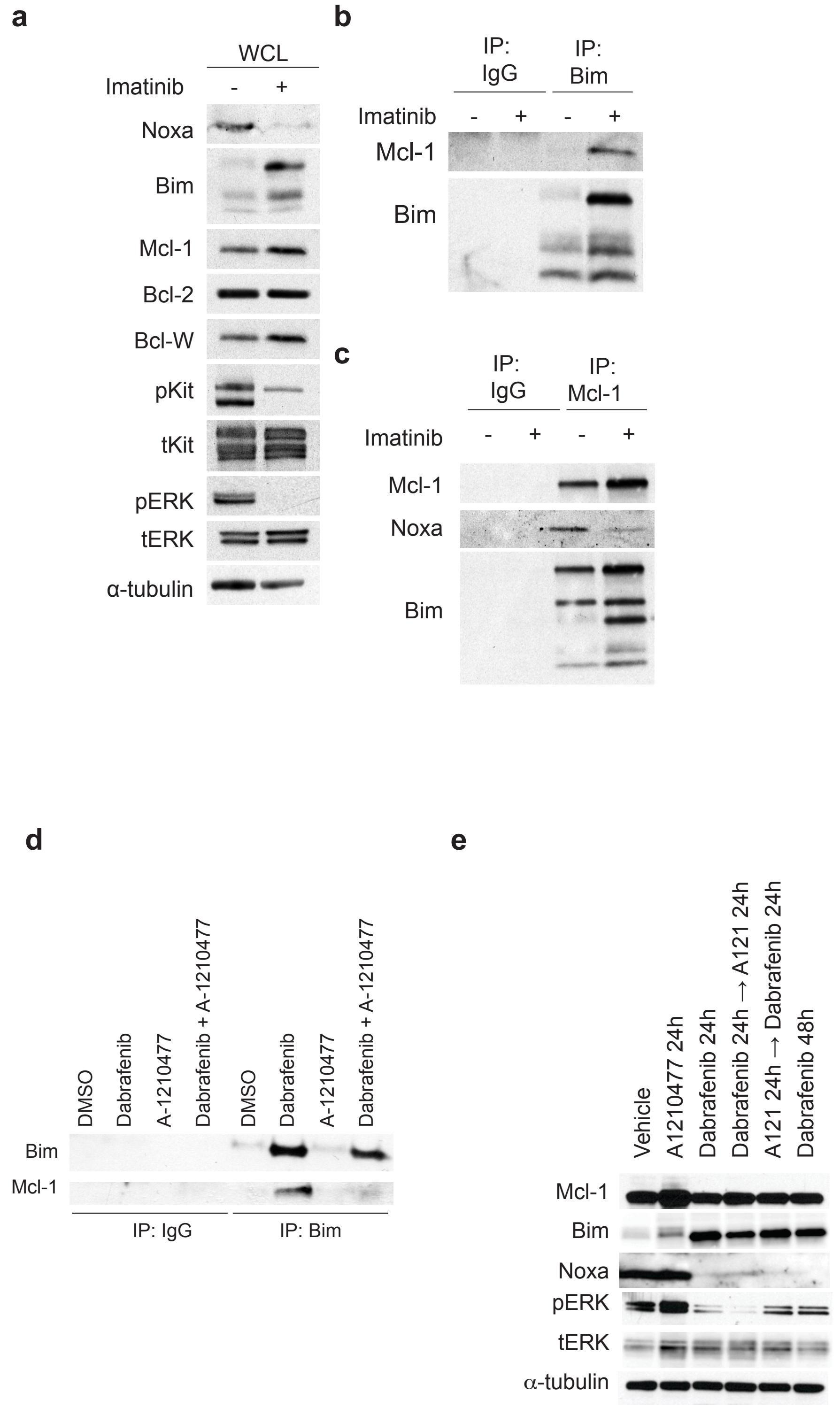

Supplementary Figure 5. (a) BH3 profiling of A375M melanoma xenografts (n = 2) following treatment with vemurafenib *in vivo*. P-values were calculated using ANOVA test followed with multiple comparison correction using Dunnett's test (*, adjusted P-value <0.05; **, adjusted P-value < 0.005; ***, adjusted P-value < 0.0005). (b) Immunoprecipitation of BIM followed by Western blot of NOXA, BIM and MCL1 in A375M xenografts after 7 hour treatment with dabrafenib or vehicle (3 tumors each). (c) Spaghetti blots showing tumor volume after administration of BRAF inhibitor, MCL1 inhibitor S63845 or sequential administration of BRAF inhibitor followed by MCL1 inhibitors. Error bars indicate mean ± s.e.m of indicated replicates. Source data for all Western blots are provided as a Source Data file.

Supplemental Figure 5

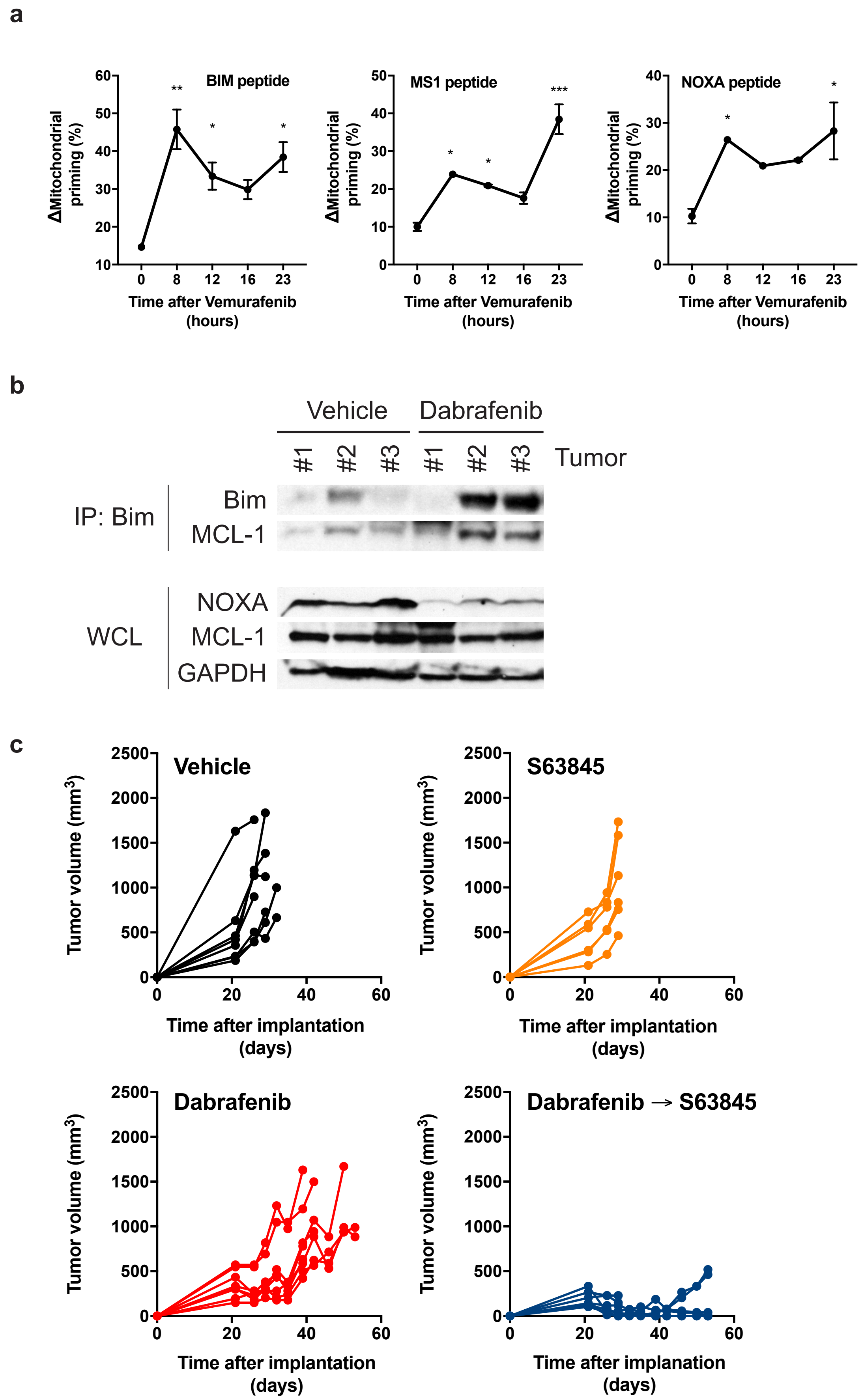

Supplementary Tables

Supplementary Table 1. (a) Cell lines used in this study along with their driver oncogene.

Supplementary Table 2. Small molecules used in this study and their oncogenic target. Final concentration of the drug used is indicated.

Supplementary Table 3. Characteristics of patient samples used in this study.

Supplementary Table 1: Cell lines used in this study

Cell line	Lineage	Driver Oncoprotein
A375M	Melanoma	BRAF
WM1575	Melanoma	BRAF
UACC-62	Melanoma	BRAF
WM1745	Melanoma	BRAF
WM451Lu	Melanoma	BRAF
SK-MEL-28	Melanoma	BRAF
COLO201	Colon adenocarcinoma	BRAF
WiDr	Rectosigmoid adenocarcinoma	BRAF
GIST48	Gastrointestinal stromal tumor	KIT
GIST882	Gastrointestinal stromal tumor	KIT
CGTH-W-1	Thyroid gland squamous cell carcinoma	KIT
NCI-H1975	Lung adenocarcinoma	EGFR
PC9	Lung adenocarcinoma	EGFR
EBC-1	Squamous cell lung carcinoma	MET
GTL-16	Gastric adenocarcinoma	MET
Kato II	Signet ring cell gastric adenocarcinoma	ALK
NCI-H2288	Non-small cell lung cancer	ALK
NCI-H1322	Non-small cell lung cancer	ALK
SHSY5Y	Neuroblastoma	ERBB2
BT-474	Invasive ductal carcinoma	ERBB2
SKBR-3	Breast adenocarcinoma	ERBB2

Supplementary Table 2: Kinase inhibitors used in this study

Drug	Target	In vitro dose (unless otherwise reported in text)
PLX4720	BRAF	1µM
Dabrafenib	BRAF	1µM
Vemurafenib	BRAF	1µM
SB590885	BRAF	1µM
Selumetinib	MEK	100nM
PD0325901	MEK	10nM
PD98059	MEK	100nM
Trametinib	MEK	100nM
PD184352	MEK	100nM
imatinib	KIT	1µM
geftinib	EGFR	1µM
crizotinib	MET, ALK	1µM
laptinib	ERBB2	1µM
AZD5991	MCL1	1µM
A1210477	MCL1	10µM
PI-103	PI3K	1µM

Supplementary Table 3
Primary Patient Samples

Identifier	Age	Sex	Primary Location	Biopsy location	Primary or Metastatic	Mutation	Prior treatment	Comments
CY98	47	F	L knee	(L) thigh soft tissue metastasis	Metastatic	BRAF V600E	s/p IFN, s/p Ipilimumab+GMCSF	
CY101	83	M	Unknown; Stage IV	Spleen	Metastatic	NRAS Q61R	s/p Ipilimumab, s/p Pembrolizumab	
CY102	72	F	Vaginal/mucosal	sample from fragmented pieces of (R) adrenal gland metastasis	Metastatic	BRAF wt, NRAS wt, NF1 wt	Nivolumab + Ipilimumab	Adrenal lesion progressing on nivo/Ipi with other lesions responding
