# Supplementary material for: Destabilization of NOXA mRNA as a common resistance mechanism to targeted therapies
Source: Nat Commun. 2019 Nov 14;10:5157. doi: 10.1038/s41467-019-12477-y (PMC6856172; doi:10.1038/s41467-019-12477-y)
Supplement: Supplementary file 3 — Reporting Summary [file 41467_2019_12477_MOESM3_ESM.pdf]

nature research

Corresponding author(s): Rizwan Haq, David E. Fisher

Last updated by author(s): 7/1/2019

Reporting Summary

Nature Research wishes to improve the reproducibility of the work that we publish. This form provides structure for consistency and transparency in reporting. For further information on Nature Research policies, see Authors & Referees and the Editorial Policy Checklist.

Statistics

For all statistical analyses, confirm that the following items are present in the figure legend, table legend, main text, or Methods section.

n/a	Confirmed	
☐	☒	The exact sample size (n) for each experimental group/condition, given as a discrete number and unit of measurement
☐	☒	A statement on whether measurements were taken from distinct samples or whether the same sample was measured repeatedly
☐	☒	The statistical test(s) used AND whether they are one- or two-sided<br>*Only common tests should be described solely by name; describe more complex techniques in the Methods section.*
☒	☐	A description of all covariates tested
☐	☒	A description of any assumptions or corrections, such as tests of normality and adjustment for multiple comparisons
☒	☐	A full description of the statistical parameters including central tendency (e.g. means) or other basic estimates (e.g. regression coefficient) AND variation (e.g. standard deviation) or associated estimates of uncertainty (e.g. confidence intervals)
☒	☐	For null hypothesis testing, the test statistic (e.g. F, t, r) with confidence intervals, effect sizes, degrees of freedom and P value noted<br>*Give P values as exact values whenever suitable.*
☒	☐	For Bayesian analysis, information on the choice of priors and Markov chain Monte Carlo settings
☒	☐	For hierarchical and complex designs, identification of the appropriate level for tests and full reporting of outcomes
☒	☐	Estimates of effect sizes (e.g. Cohen's d, Pearson's r), indicating how they were calculated

Our web collection on statistics for biologists contains articles on many of the points above.

Software and code

Policy information about availability of computer code

Data collection	not applicable
Data analysis	Data analysis was performed in Microsoft Excel v16. Statistical analysis and graph generation was done using GraphPad Prism 8.

For manuscripts utilizing custom algorithms or software that are central to the research but not yet described in published literature, software must be made available to editors/reviewers. We strongly encourage code deposition in a community repository (e.g. GitHub). See the Nature Research guidelines for submitting code & software for further information.

Data

Policy information about availability of data

All manuscripts must include a data availability statement. This statement should provide the following information, where applicable:

- Accession codes, unique identifiers, or web links for publicly available datasets
- A list of figures that have associated raw data
- A description of any restrictions on data availability

Raw, uncropped Western blots are provided as a Source Data File. All relevant data can be inquired from the authors.

Field-specific reporting

Please select the one below that is the best fit for your research. If you are not sure, read the appropriate sections before making your selection.

☒ Life sciences ☐ Behavioural & social sciences ☐ Ecological, evolutionary & environmental sciences

For a reference copy of the document with all sections, see nature.com/documents/nr-reporting-summary-flat.pdf

Life sciences study design

All studies must disclose on these points even when the disclosure is negative.

Sample size	No sample size calculation was performed beforehand. Sample sizes were chosen according to established practice in the field.
Data exclusions	No data point has been excluded.
Replication	For reproducibility biological replicates were performed on different days.
Randomization	n/a
Blinding	n/a

Reporting for specific materials, systems and methods

We require information from authors about some types of materials, experimental systems and methods used in many studies. Here, indicate whether each material, system or method listed is relevant to your study. If you are not sure if a list item applies to your research, read the appropriate section before selecting a response.

Materials & experimental systems

n/a	Involved in the study	
☐	☒	Antibodies
☐	☒	Eukaryotic cell lines
☒	☐	Palaeontology
☐	☒	Animals and other organisms
☐	☒	Human research participants
☒	☐	Clinical data

Methods

n/a	Involved in the study	
☒	☐	ChIP-seq
☐	☒	Flow cytometry
☒	☐	MRI-based neuroimaging

Antibodies

Antibodies used	The following primary antibodies were used for western blot: mouse anti-NOXA (Enzo Life Sciences, ALX-804-408), rabbit anti-Bcl-2 (Cell Signaling Technology, #2870 and #15071), rabbit anti-Bcl-w (CST, #2724), rabbit anti-Bcl-xl (BD Biosciences, #610211), rabbit anti-Mcl-1 (CST, #4572), rabbit anti-Bim (CST, #2933), rabbit anti-phospho-EGFR (Y1068) (CST, #2234), rabbit anti-EGFR (CST, #4267), rabbit anti-phospho-HER2 (Y1248) (CST, #2243), rabbit anti-HER2 (CST, #2165), rabbit anti-phospho-c-KIT Y719 (CST, #3391), rabbit anti-c-KIT (CST, #3074), rabbit anti-phospho-ALK (CST, #3341), rabbit anti-ALK (CST, #3633), rabbit anti-phospho-MET Y1349 (CST, #3121), rabbit anti-MET (CST, #8198), rabbit anti-phospho-ERK (CST, #9101), rabbit anti-ERK (CST, #4695), mouse anti-alpha- tubulin (Sigma-Aldrich, T9026), vinculin (Abcam ab129002), Cleaved PARP CST #5625). The following HRP-linked secondary antibodies were used: horse anti-mouse-IgG (CST, #7076), goat anti-rabbit-IgG (CST, #7074), and mouse anti-rabbit-IgG (conformation specific) (CST, #5127). Anti-V5 antibody or affinity gel was obtained from Thermo-Fisher Scientific. The following antibodies were used for immunoprecipitation: mouse anti-Mcl-1 (BD, #559027), rabbit anti-Bim (CST, #2933), normal mouse IgG (Santa Cruz, #2025), normal rabbit IgG (Santa Cruz, #2027), Protein A magnetic beads (CST, #8687), Protein G magnetic beads (CST, #8740).
Validation	Only commercial and validated antibodies have been used.

Eukaryotic cell lines

Policy information about cell lines

Cell line source(s)	Cell lines were obtained from ATCC or from the Center for Molecular Therapeutics, Mass General Hospital.
Authentication	Cells were authenticated by SITR profiles at the Center for Molecular Therapeutics, Mass General Hospital.
Mycoplasma contamination	Cells were tested for Mycoplasma and were found negative.
Commonly misidentified lines (See ICLAC register)	No commonly misidentified cell lines have been used.

Animals and other organisms

Policy information about studies involving animals; ARRIVE guidelines recommended for reporting animal research

Laboratory animals	B6.Cg-Foxn1<nu>/J were obtained from JAX laboratories or Charles River Laboratories

Wild animals	not applicable
Field-collected samples	not applicable
Ethics oversight	All experiments were performed in compliance with federal laws and institutional guidelines and were approved by the Animal Care and Use Committee of the Dana-Farber Cancer Institute.

Note that full information on the approval of the study protocol must also be provided in the manuscript.

Human research participants

Policy information about studies involving human research participants

Population characteristics	*Describe the covariate-relevant population characteristics of the human research participants (e.g. age, gender, genotypic information, past and current diagnosis and treatment categories). If you filled out the behavioural & social sciences study design questions and have nothing to add here, write "See above."*
Recruitment	Biopsies and cell lines were obtained from patients with large melanomas (those who required surgical intervention)
Ethics oversight	All patients gave informed consent for tissue acquisition as per IRB-approved protocol 05-042 or 11-181 (Office for Human Research Studies, Dana-Farber/Harvard Cancer Center).

Note that full information on the approval of the study protocol must also be provided in the manuscript.

Flow Cytometry

Plots

Confirm that:

☒ The axis labels state the marker and fluorochrome used (e.g. CD4-FITC).

☒ The axis scales are clearly visible. Include numbers along axes only for bottom left plot of group (a 'group' is an analysis of identical markers).

☒ All plots are contour plots with outliers or pseudocolor plots.

☒ A numerical value for number of cells or percentage (with statistics) is provided.

Methodology

Sample preparation	Refer to methods section for details of sample preparation and staining.
Instrument	BD FACS Fortessa or similar
Software	BD FacsDiva
Cell population abundance	not applicable
Gating strategy	Gating strategy is provided in Supplementary Information

☒ Tick this box to confirm that a figure exemplifying the gating strategy is provided in the Supplementary Information.
