# Supplementary material for: Destabilization of NOXA mRNA as a common resistance mechanism to targeted therapies
Source: Nat Commun. 2019 Nov 14;10:5157. doi: 10.1038/s41467-019-12477-y (PMC6856172; doi:10.1038/s41467-019-12477-y)
Supplement: Supplementary file 4 — Source Data [file 41467_2019_12477_MOESM4_ESM.zip › FIgure 2a Westerns/A375 SKMEL5 MCL1 1.pdf]

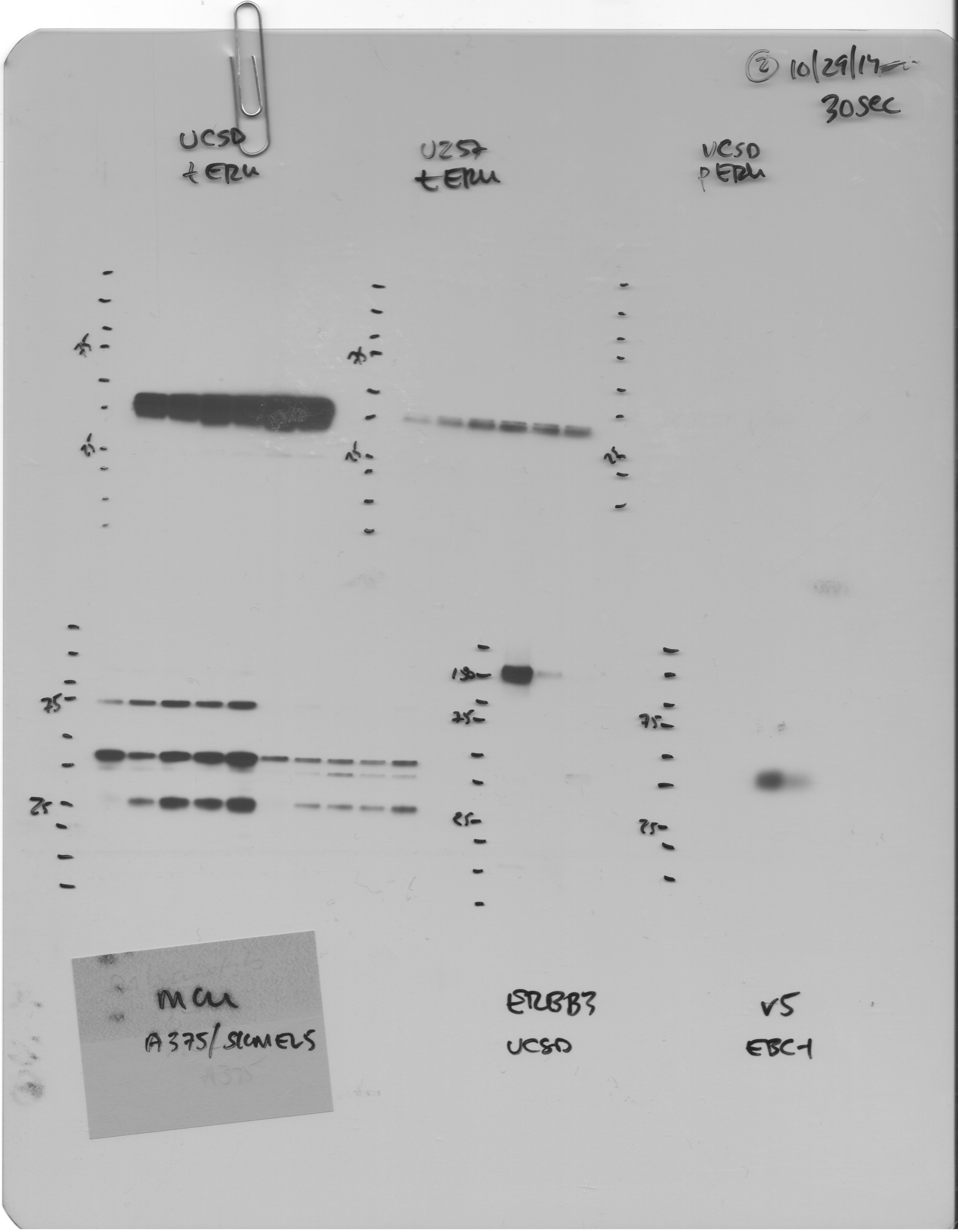
② 10/29/17
30sec
UCSD tERK
UZ57 tERK
UCSD pERK
75
25
MCM
A375/SKMEL5
ERBB3
UCSD
V5
EBC-1
