# Supplementary material for: Destabilization of NOXA mRNA as a common resistance mechanism to targeted therapies
Source: Nat Commun. 2019 Nov 14;10:5157. doi: 10.1038/s41467-019-12477-y (PMC6856172; doi:10.1038/s41467-019-12477-y)
Supplement: Supplementary file 4 — Source Data [file 41467_2019_12477_MOESM4_ESM.zip › FIgure 2a Westerns/A375 SKMEL5 Bcl2.pdf]

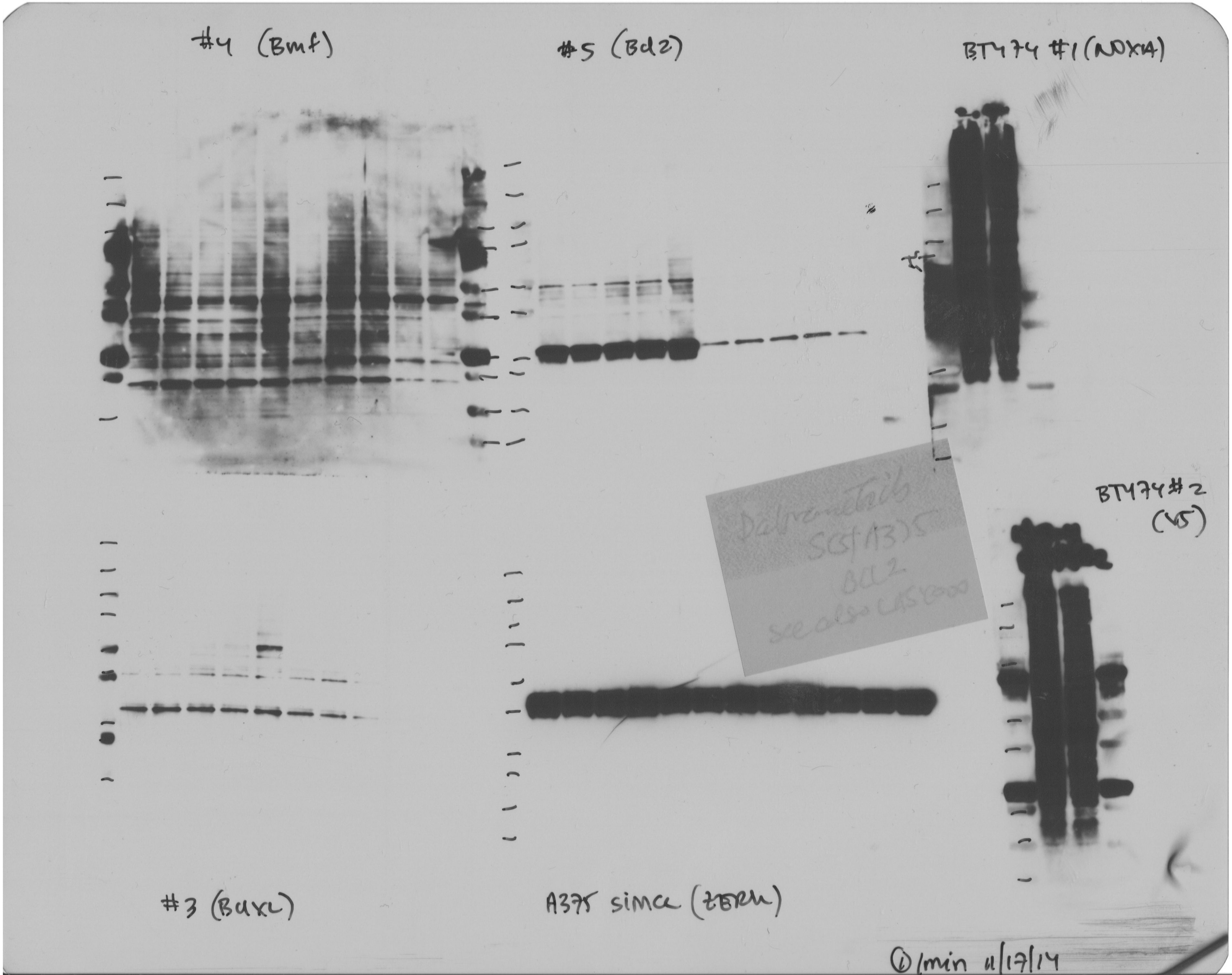

#4 (Bmf)
#5 (Bcl2)
BT474 #1 (NOXA)
BT474 #2 (V5)
Dabrafenib
BCL2
see also
#3 (BcxL)
A375 siMcl
① 1min 11/17/14
