# Supplementary material for: Destabilization of NOXA mRNA as a common resistance mechanism to targeted therapies
Source: Nat Commun. 2019 Nov 14;10:5157. doi: 10.1038/s41467-019-12477-y (PMC6856172; doi:10.1038/s41467-019-12477-y)
Supplement: Supplementary file 4 — Source Data [file 41467_2019_12477_MOESM4_ESM.zip › FIgure 2a Westerns/A375 SKMEL5 Bcl-w.pdf]

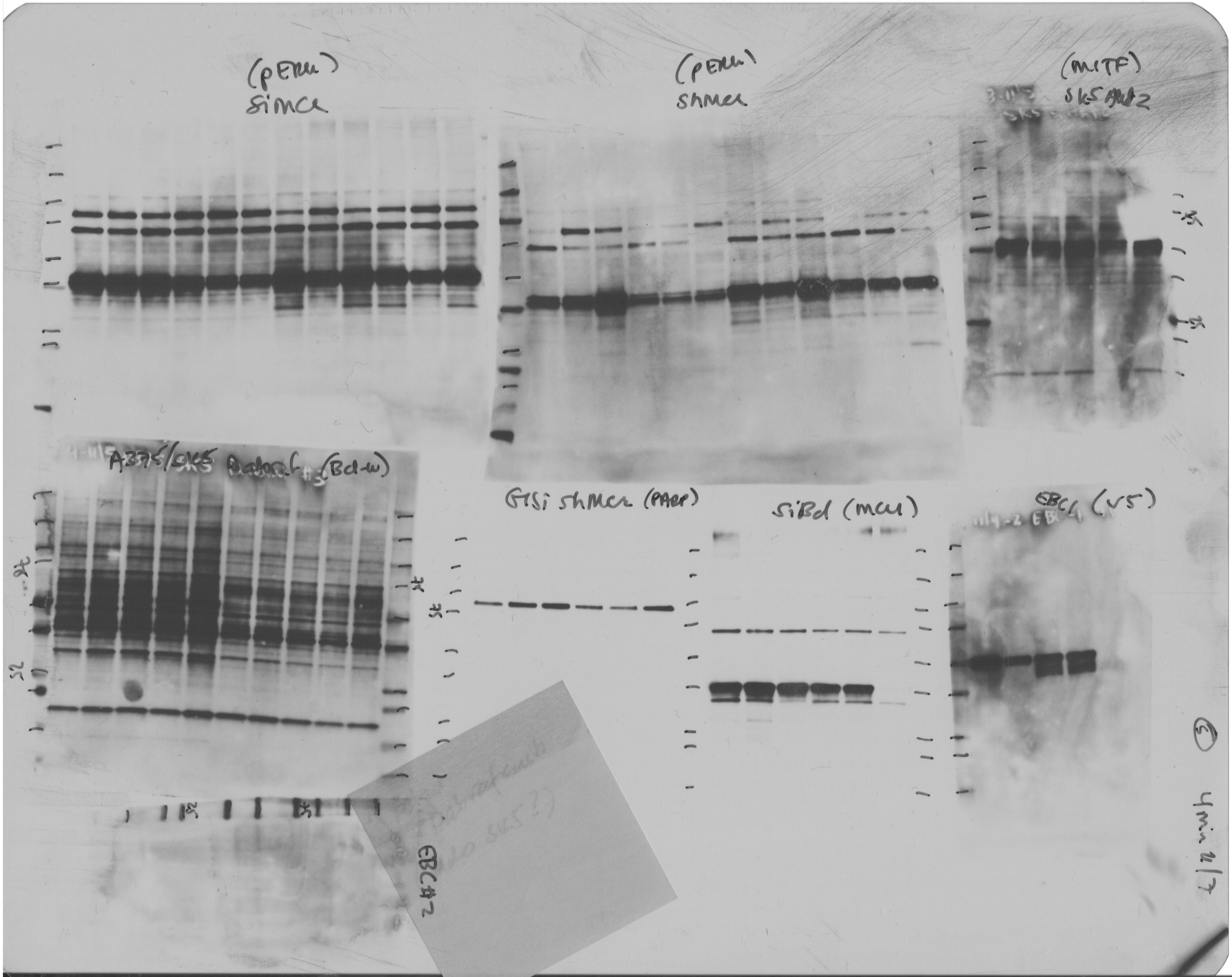
(pERK)
siMcl
(pERK)
shMcl
(MITF)
(Bcl-w)
shMcl (PARP)
siBcl (Mcl)
(V5)
EBC#2
4min 11/7
