# Supplementary material for: Destabilization of NOXA mRNA as a common resistance mechanism to targeted therapies
Source: Nat Commun. 2019 Nov 14;10:5157. doi: 10.1038/s41467-019-12477-y (PMC6856172; doi:10.1038/s41467-019-12477-y)

① 1min
12/19/14

Bmf

A375/S165 Dabraf R #7

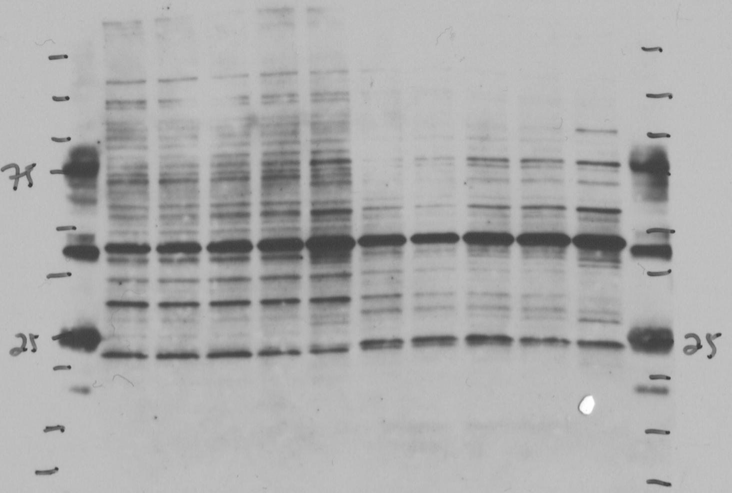

Supplement: Supplementary file 4 — Source Data [file 41467_2019_12477_MOESM4_ESM.zip › FIgure 2a Westerns/A375 SKMEL5 Bmf.pdf]
