# Supplementary material for: Destabilization of NOXA mRNA as a common resistance mechanism to targeted therapies
Source: Nat Commun. 2019 Nov 14;10:5157. doi: 10.1038/s41467-019-12477-y (PMC6856172; doi:10.1038/s41467-019-12477-y)
Supplement: Supplementary file 4 — Source Data [file 41467_2019_12477_MOESM4_ESM.zip › FIgure 2a Westerns/A375 SKMEL5 Puma.pdf]

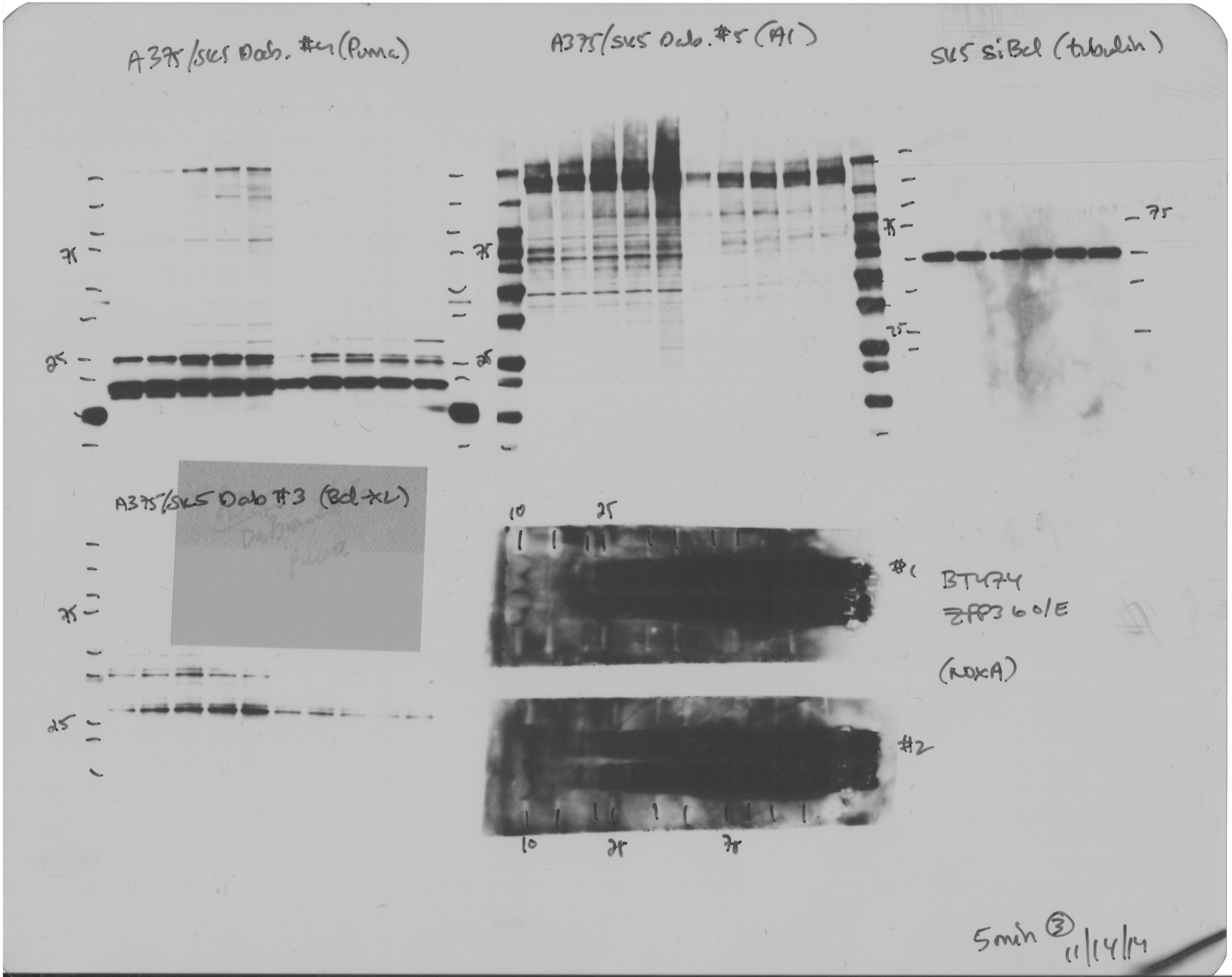
A375/SK5 Dab. #4 (Puma)
A375/SK5 Dab. #5 (A1)
SK5 siBcl (tubulin)
A375/SK5 Dab #3 (Bcl-xL)
#1
BT474
ZFP360/E
(NOXA)
#2
5min ③
11/14/14
