# Supplementary material for: Destabilization of NOXA mRNA as a common resistance mechanism to targeted therapies
Source: Nat Commun. 2019 Nov 14;10:5157. doi: 10.1038/s41467-019-12477-y (PMC6856172; doi:10.1038/s41467-019-12477-y)
Supplement: Supplementary file 4 — Source Data [file 41467_2019_12477_MOESM4_ESM.zip › FIgure 2a Westerns/SKMEL5 A375 NOXA.pdf]

~~A375/SK5~~

② 30sec.

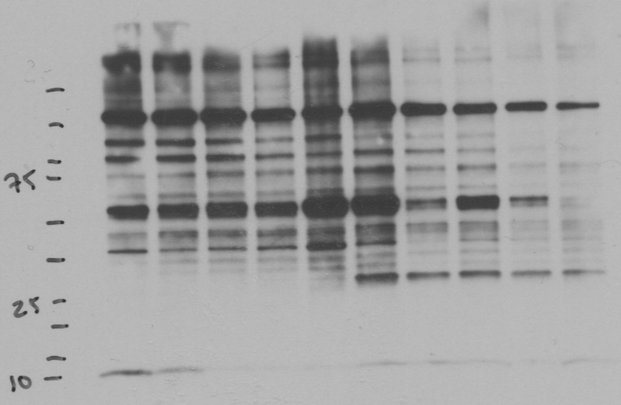

A375/SK5
Dabraf #8 (2/4)

NOXA

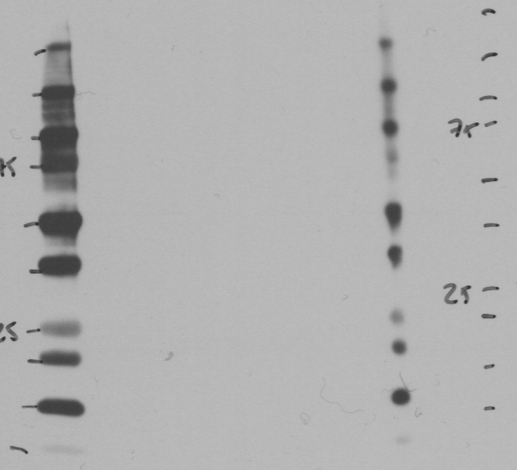

pkir #2   tkir #3

GST-T1 mcl1 +LDm (2/4)
