# Supplementary material for: Destabilization of NOXA mRNA as a common resistance mechanism to targeted therapies
Source: Nat Commun. 2019 Nov 14;10:5157. doi: 10.1038/s41467-019-12477-y (PMC6856172; doi:10.1038/s41467-019-12477-y)
Supplement: Supplementary file 4 — Source Data [file 41467_2019_12477_MOESM4_ESM.zip › FIgure 2a Westerns/A375 SKMEL5 A1.pdf]

A375/SK5 Dabral TC

#4

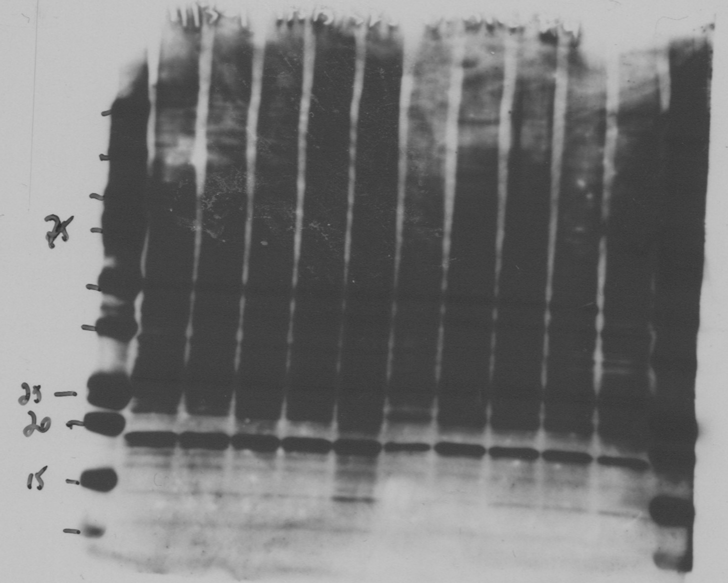

BclXL #4
(30kd)

#5

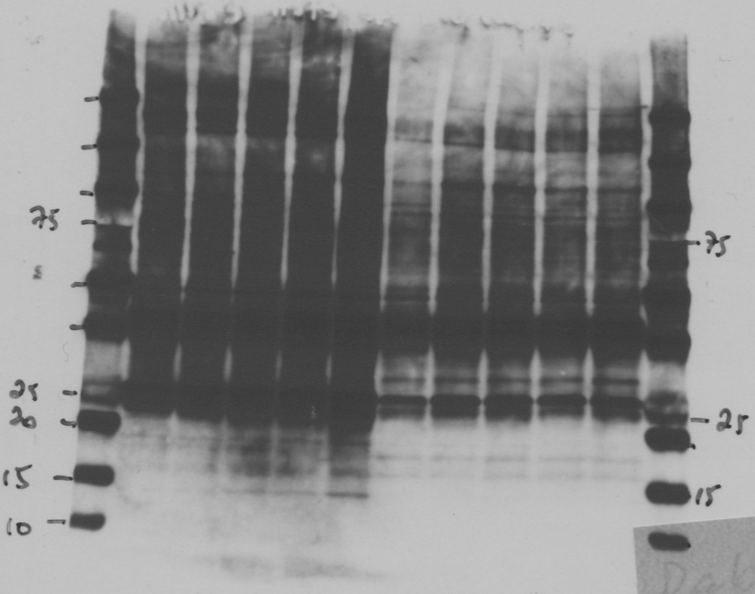

#5
A1
(18kd)

Dabrafenib
SKT
A1
lower?

① 1min 11/19/14
