# Supplementary material for: Destabilization of NOXA mRNA as a common resistance mechanism to targeted therapies
Source: Nat Commun. 2019 Nov 14;10:5157. doi: 10.1038/s41467-019-12477-y (PMC6856172; doi:10.1038/s41467-019-12477-y)

(1) 1min
9/16/15

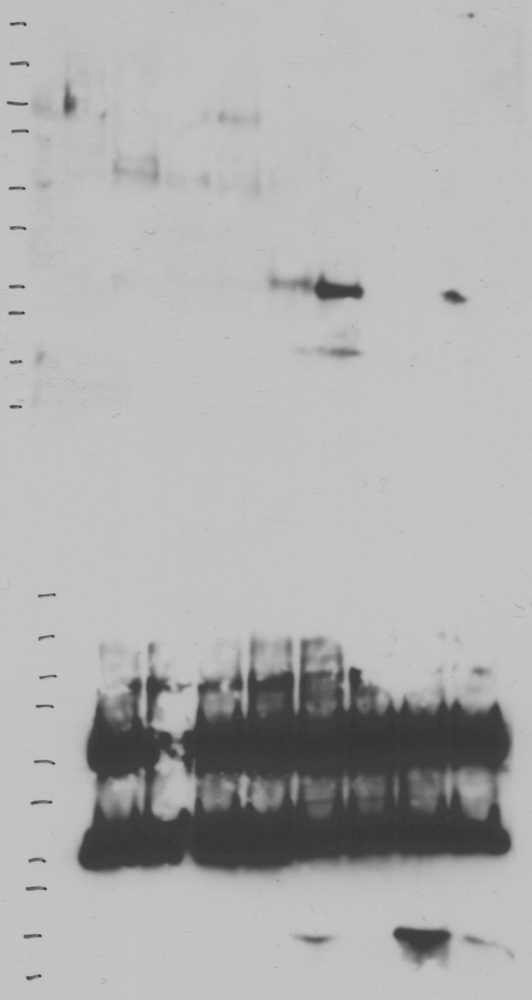

9/15 -
A375 Bim IP #2
(Bim + 2° cont. sp.)

9/5 -
A375 MCU IP #2
(NOXA, ms)

A375 MCU IP
DS
to reprobe

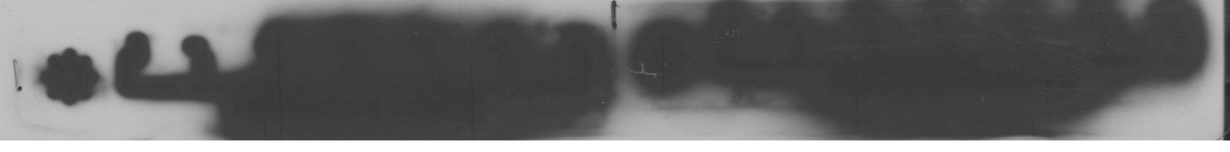

Supplement: Supplementary file 4 — Source Data [file 41467_2019_12477_MOESM4_ESM.zip › Figure 4e Westerns/Figure 4e IP MCL1 W NOXA.pdf]
