# Supplementary material for: Destabilization of NOXA mRNA as a common resistance mechanism to targeted therapies
Source: Nat Commun. 2019 Nov 14;10:5157. doi: 10.1038/s41467-019-12477-y (PMC6856172; doi:10.1038/s41467-019-12477-y)
Supplement: Supplementary file 4 — Source Data [file 41467_2019_12477_MOESM4_ESM.zip › Figure 4d Westerns/Figure 4d MCL1 WCL.pdf]

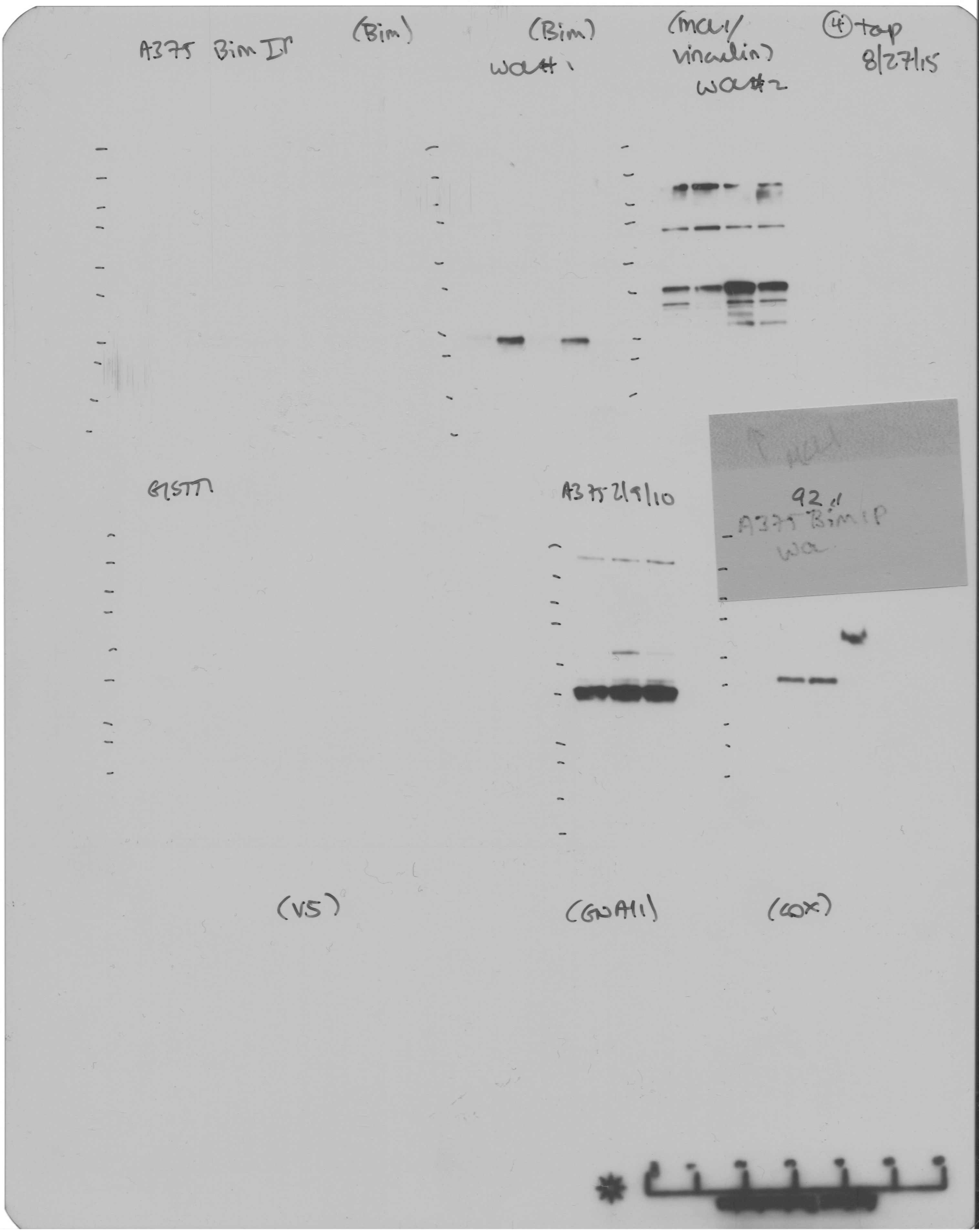
A375 Bim IP
(Bim)
(Bim)
wash 1
(mcl1/
vinculin)
wash 2
4 top
8/27/15
GSTT1
A375 2/9/10
92 11
A375 Bim IP
wa
(V5)
(GNA11)
(cox)
