# Supplementary material for: Destabilization of NOXA mRNA as a common resistance mechanism to targeted therapies
Source: Nat Commun. 2019 Nov 14;10:5157. doi: 10.1038/s41467-019-12477-y (PMC6856172; doi:10.1038/s41467-019-12477-y)
Supplement: Supplementary file 4 — Source Data [file 41467_2019_12477_MOESM4_ESM.zip › Figure 4d Westerns/Figure 4d Bim WCL.pdf]

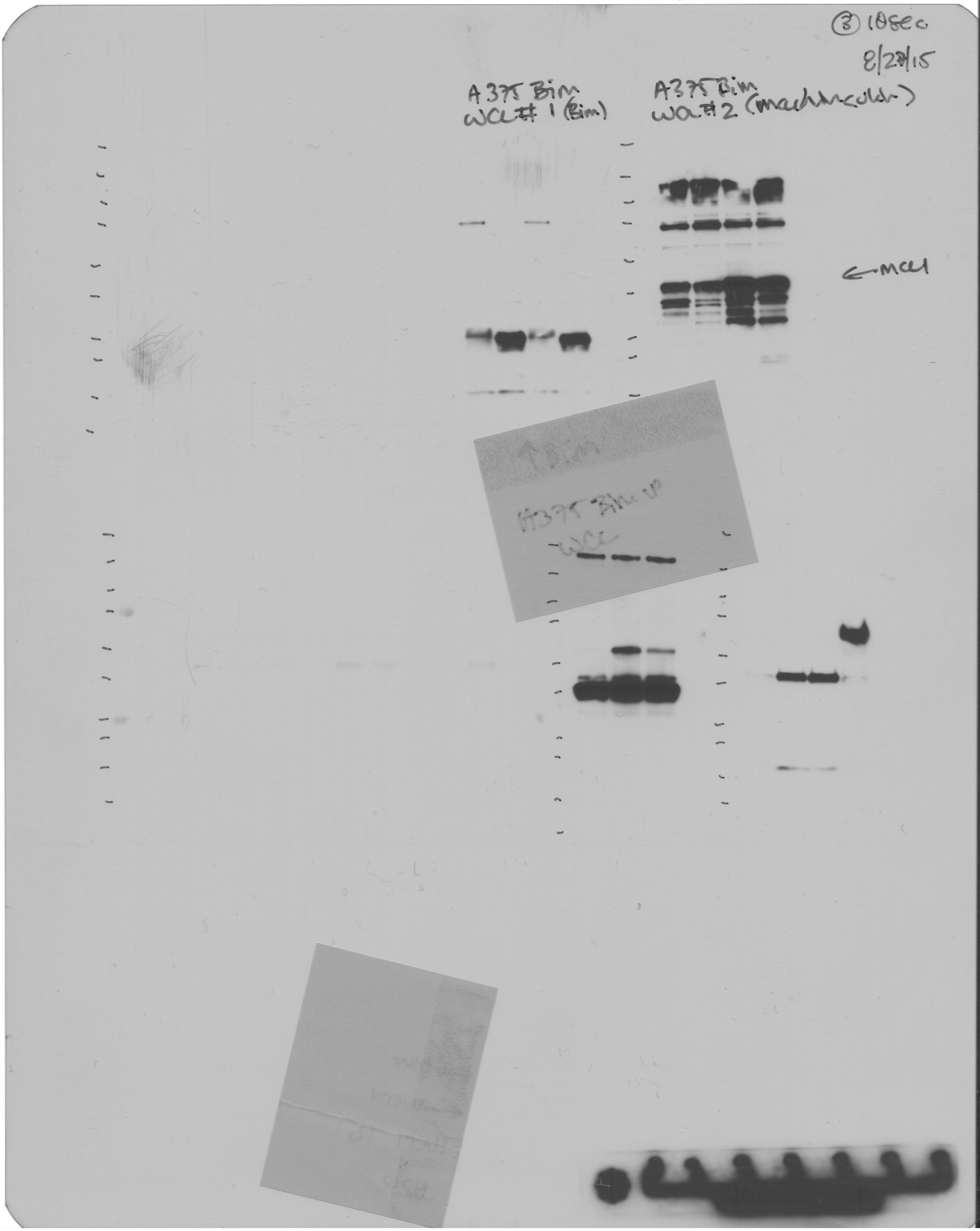
③ 10sec
8/27/15
A375 Bim
WCL#1 (Bim)
A375 Bim
WCL#2
← Mcl1
↑ Bim
A375 Bim IP
WCL
