# Supplementary material for: Destabilization of NOXA mRNA as a common resistance mechanism to targeted therapies
Source: Nat Commun. 2019 Nov 14;10:5157. doi: 10.1038/s41467-019-12477-y (PMC6856172; doi:10.1038/s41467-019-12477-y)
Supplement: Supplementary file 4 — Source Data [file 41467_2019_12477_MOESM4_ESM.zip › Figure 4d Westerns/Figure 4d Vinculin WCL.pdf]

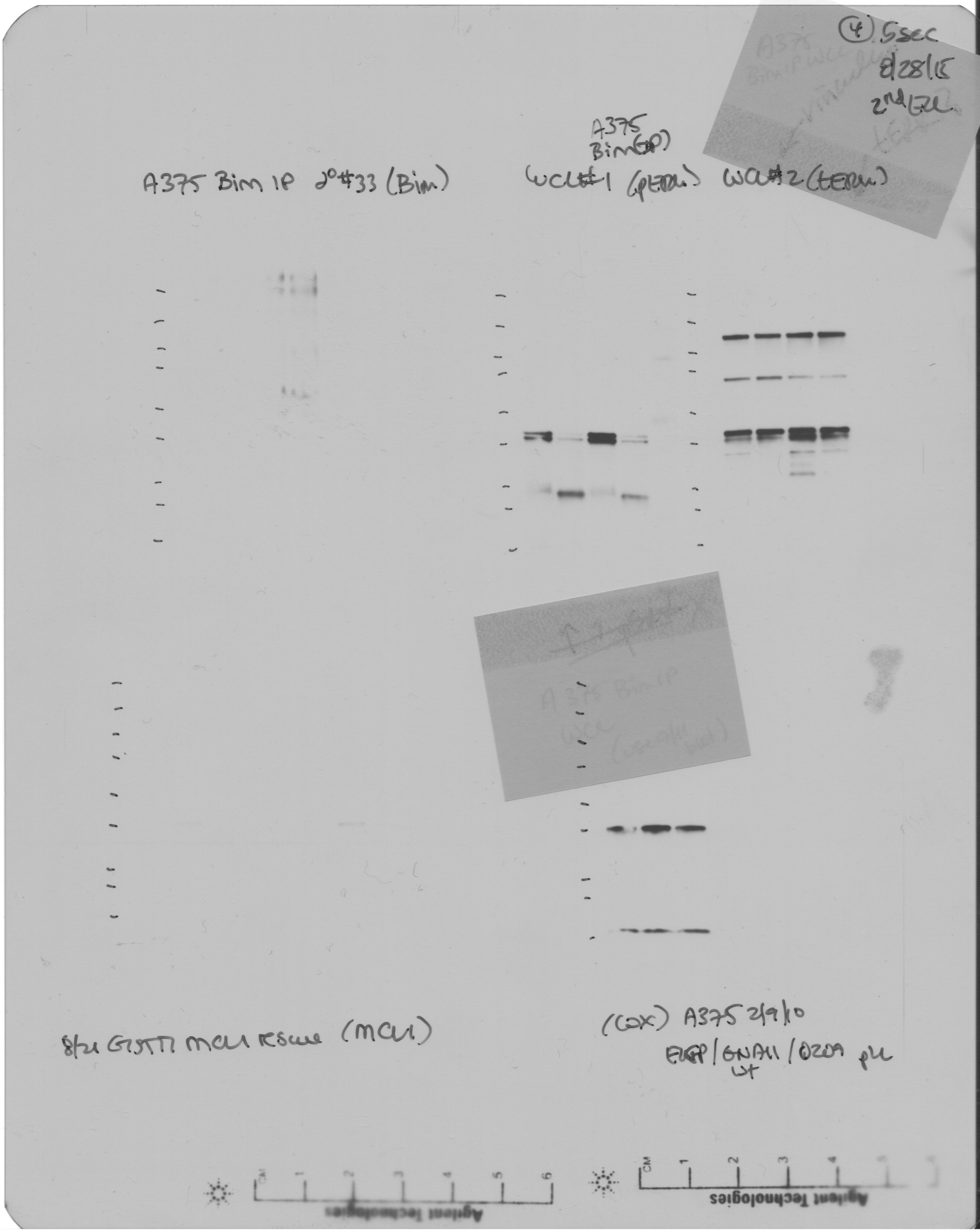

④ 5sec
8/28/15
2nd ECL
A375 Bim IP 2°#33 (Bim)
A375 Bim(IP)
WCL#1 (pERK)
WCL#2 (tERK)
A375 Bim IP WCL
8/21 G155T1 MCL1 rescue (MCL1)
(COX) A375 2/9/10
EGFP / GNA11 WT / Q209 pLL
Agilent Technologies
