# Supplementary material for: Destabilization of NOXA mRNA as a common resistance mechanism to targeted therapies
Source: Nat Commun. 2019 Nov 14;10:5157. doi: 10.1038/s41467-019-12477-y (PMC6856172; doi:10.1038/s41467-019-12477-y)
Supplement: Supplementary file 4 — Source Data [file 41467_2019_12477_MOESM4_ESM.zip › Figure 4d Westerns/Figure 4d pERK tERK WCL.pdf]

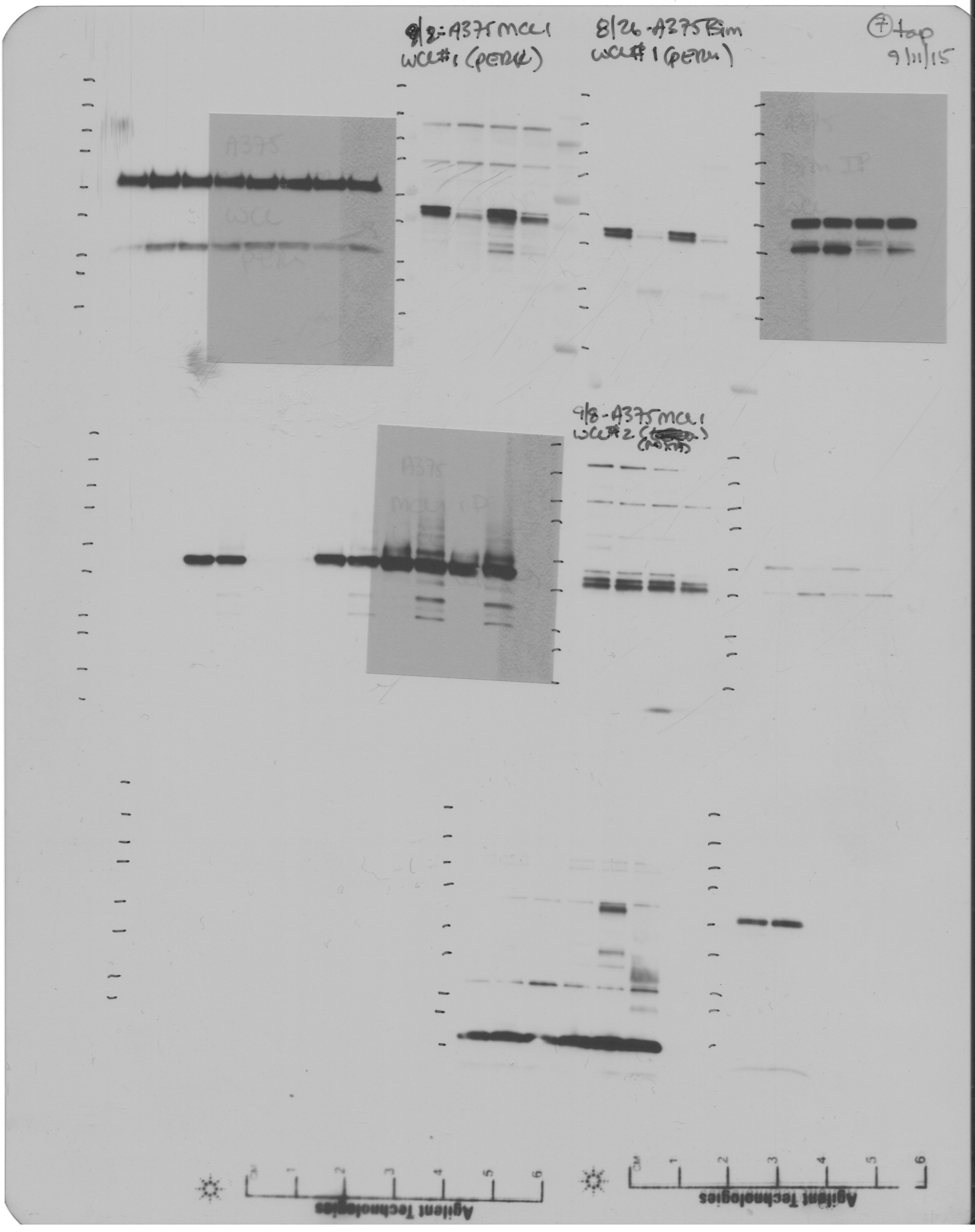
9/2 - A375 MCL1
WCL#1 (pERK)
8/26 - A375 Bim
WCL#1 (pERK)
7 top
9/11/15
A375
WCL
pERK
9/8 - A375 MCL1
WCL#2
A375
MCL1 IP
A375
Bim IP
