# Supplementary material for: Destabilization of NOXA mRNA as a common resistance mechanism to targeted therapies
Source: Nat Commun. 2019 Nov 14;10:5157. doi: 10.1038/s41467-019-12477-y (PMC6856172; doi:10.1038/s41467-019-12477-y)
Supplement: Supplementary file 4 — Source Data [file 41467_2019_12477_MOESM4_ESM.zip › Figure 4d Westerns/Figure 4d NOXA WCL.pdf]

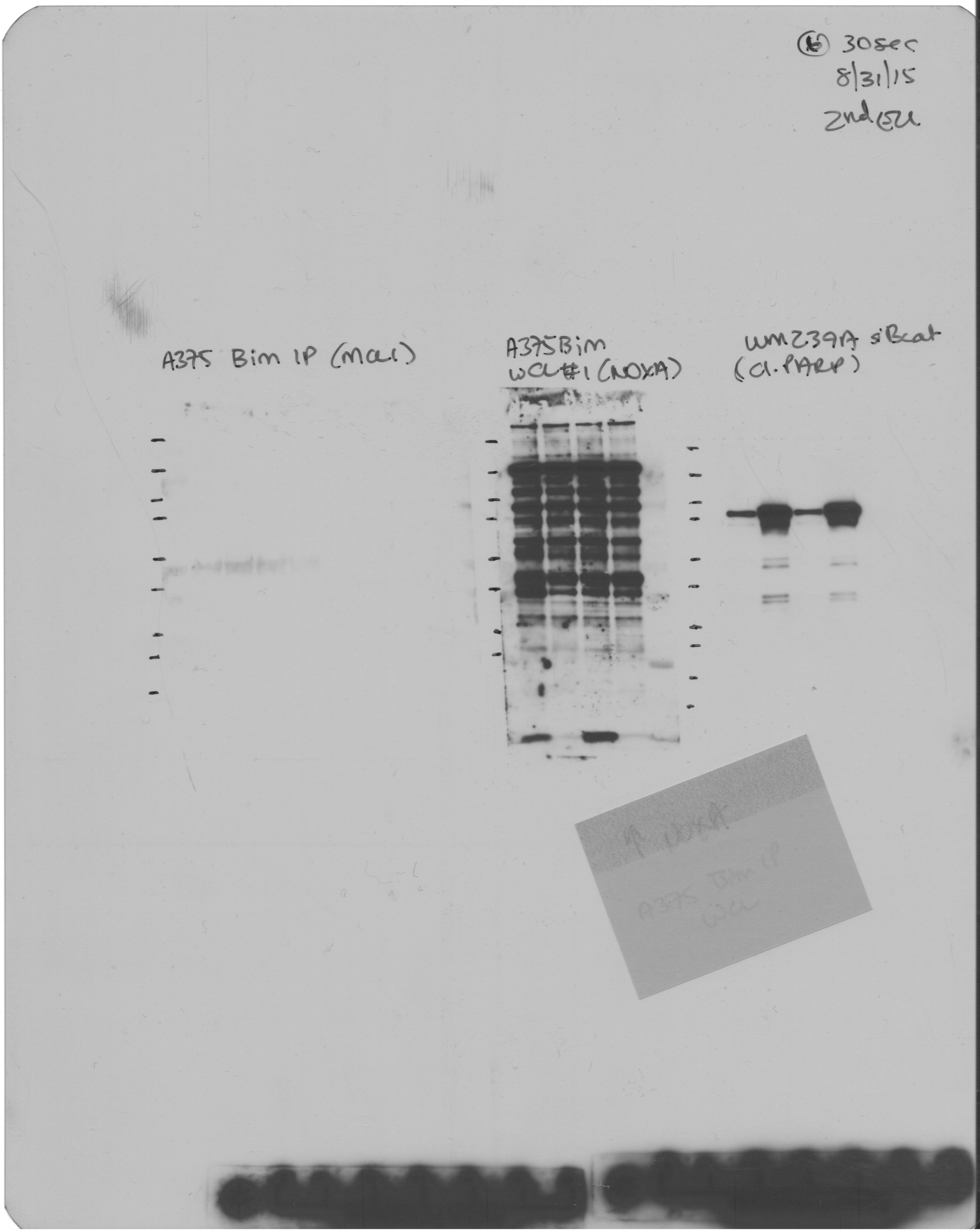
30sec
8/31/15
2nd ECL
A375 Bim IP (Mcl1)
A375 Bim
WCL#1 (NOXA)
WM239A siBcat
(cl. PARP)
↑ NOXA
A375 Bim IP
WCL
