# Supplementary material for: Destabilization of NOXA mRNA as a common resistance mechanism to targeted therapies
Source: Nat Commun. 2019 Nov 14;10:5157. doi: 10.1038/s41467-019-12477-y (PMC6856172; doi:10.1038/s41467-019-12477-y)
Supplement: Supplementary file 4 — Source Data [file 41467_2019_12477_MOESM4_ESM.zip › Figure 2c Westerns/Figure 2c pERK.pdf]

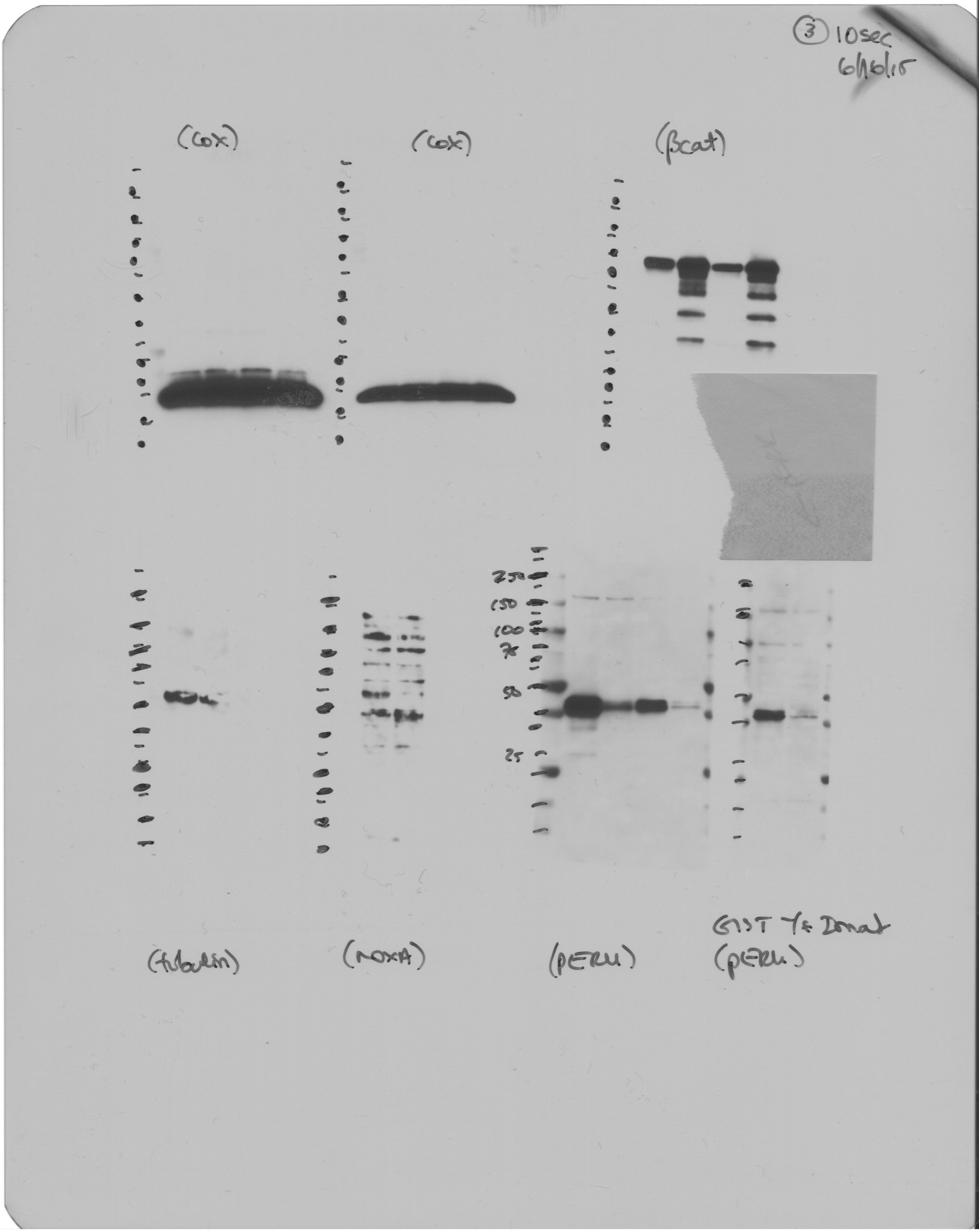
③ 10sec
6/16/15
(cox)
(cox)
(βcat)
250
150
100
75
50
25
(tubulin)
(NOXA)
(pERK)
GIST
(pERK)
