# Supplementary material for: Destabilization of NOXA mRNA as a common resistance mechanism to targeted therapies
Source: Nat Commun. 2019 Nov 14;10:5157. doi: 10.1038/s41467-019-12477-y (PMC6856172; doi:10.1038/s41467-019-12477-y)
Supplement: Supplementary file 4 — Source Data [file 41467_2019_12477_MOESM4_ESM.zip › Figure 2c Westerns/Figure 2c pKIT & COXIV.pdf]

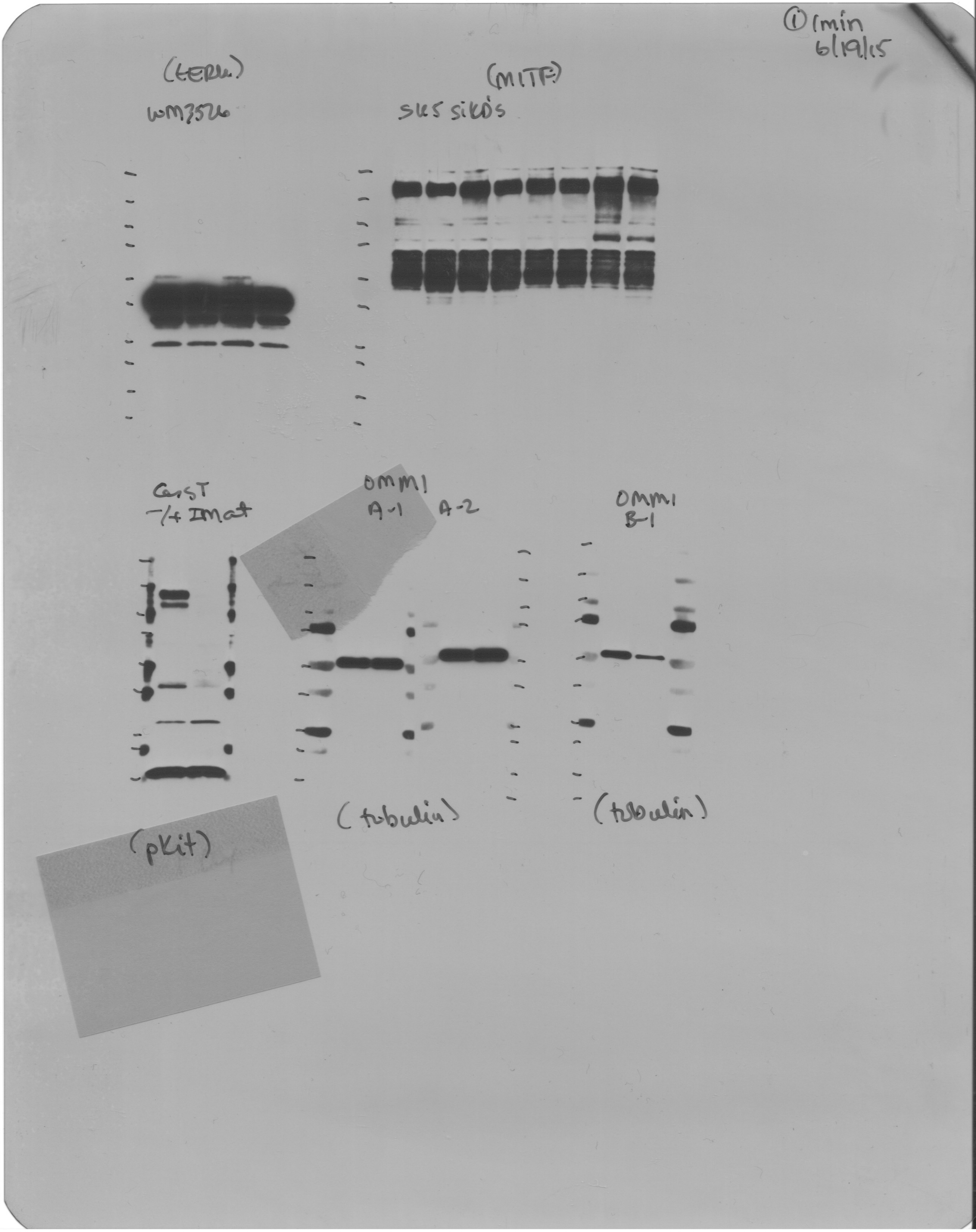

1min
6/19/15
(tERK)
WM3526
(MITF)
SK5 siKD's
GIST
−/+ Imat
OMM1
A-1 A-2
OMM1
B-1
(tubulin)
(tubulin)
(pKit)
