# Supplementary material for: Destabilization of NOXA mRNA as a common resistance mechanism to targeted therapies
Source: Nat Commun. 2019 Nov 14;10:5157. doi: 10.1038/s41467-019-12477-y (PMC6856172; doi:10.1038/s41467-019-12477-y)
Supplement: Supplementary file 4 — Source Data [file 41467_2019_12477_MOESM4_ESM.zip › Supplementary Figure 1c Westerns/Figure S1c PARP.pdf]

② 30sec 3/21/16 1st ECL

(total Akt1, rbt)

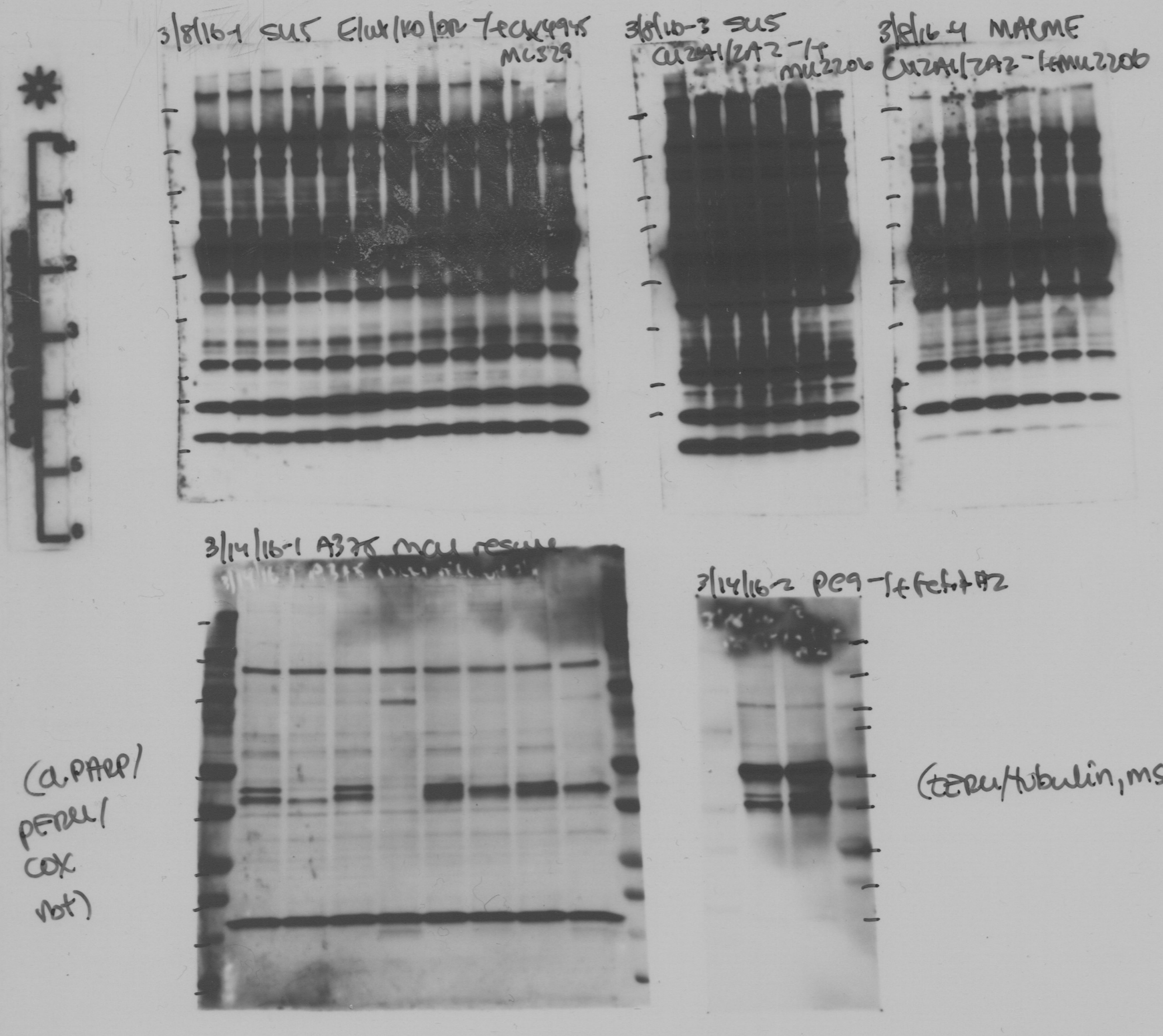

Agilent Technologies
