# Supplementary material for: Destabilization of NOXA mRNA as a common resistance mechanism to targeted therapies
Source: Nat Commun. 2019 Nov 14;10:5157. doi: 10.1038/s41467-019-12477-y (PMC6856172; doi:10.1038/s41467-019-12477-y)
Supplement: Supplementary file 4 — Source Data [file 41467_2019_12477_MOESM4_ESM.zip › Supplementary Figure 1c Westerns/Figure S1c MCL1 exposure #3.pdf]

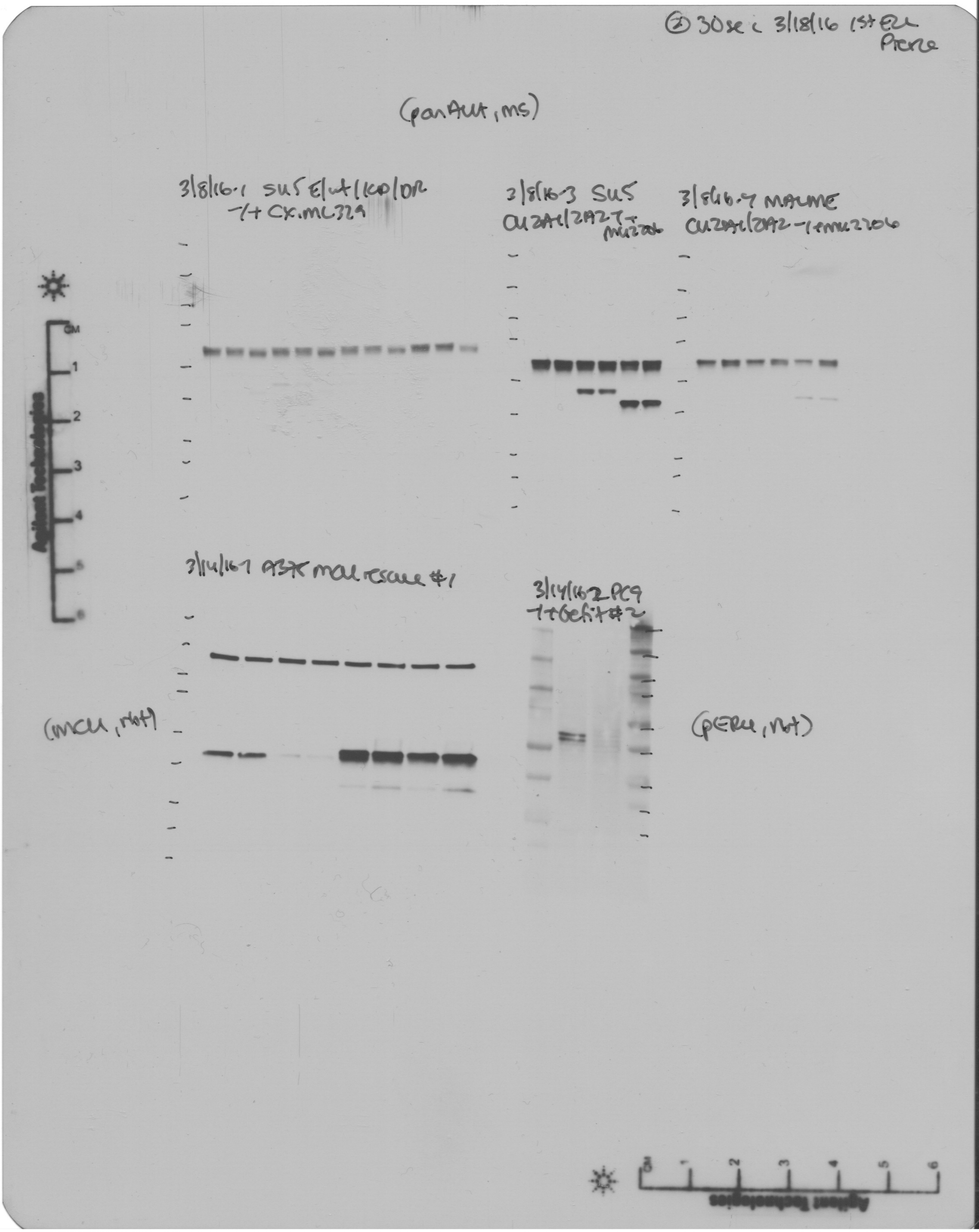
② 30sec 3/18/16 1st ECL Pierce
(panAkt, ms)
3/8/16-1 SU5
3/8/16-3 SU5
3/8/16-4 MALME
3/14/16-1 A375 MAL rescue #1
3/14/16-2 PC9
(mcl1, rbt)
(pERK, rbt)
Agilent Technologies
cm
1
2
3
4
5
6
