# Supplementary material for: Destabilization of NOXA mRNA as a common resistance mechanism to targeted therapies
Source: Nat Commun. 2019 Nov 14;10:5157. doi: 10.1038/s41467-019-12477-y (PMC6856172; doi:10.1038/s41467-019-12477-y)
Supplement: Supplementary file 4 — Source Data [file 41467_2019_12477_MOESM4_ESM.zip › Supplementary Figure 1c Westerns/Figure S1c pERK.pdf]

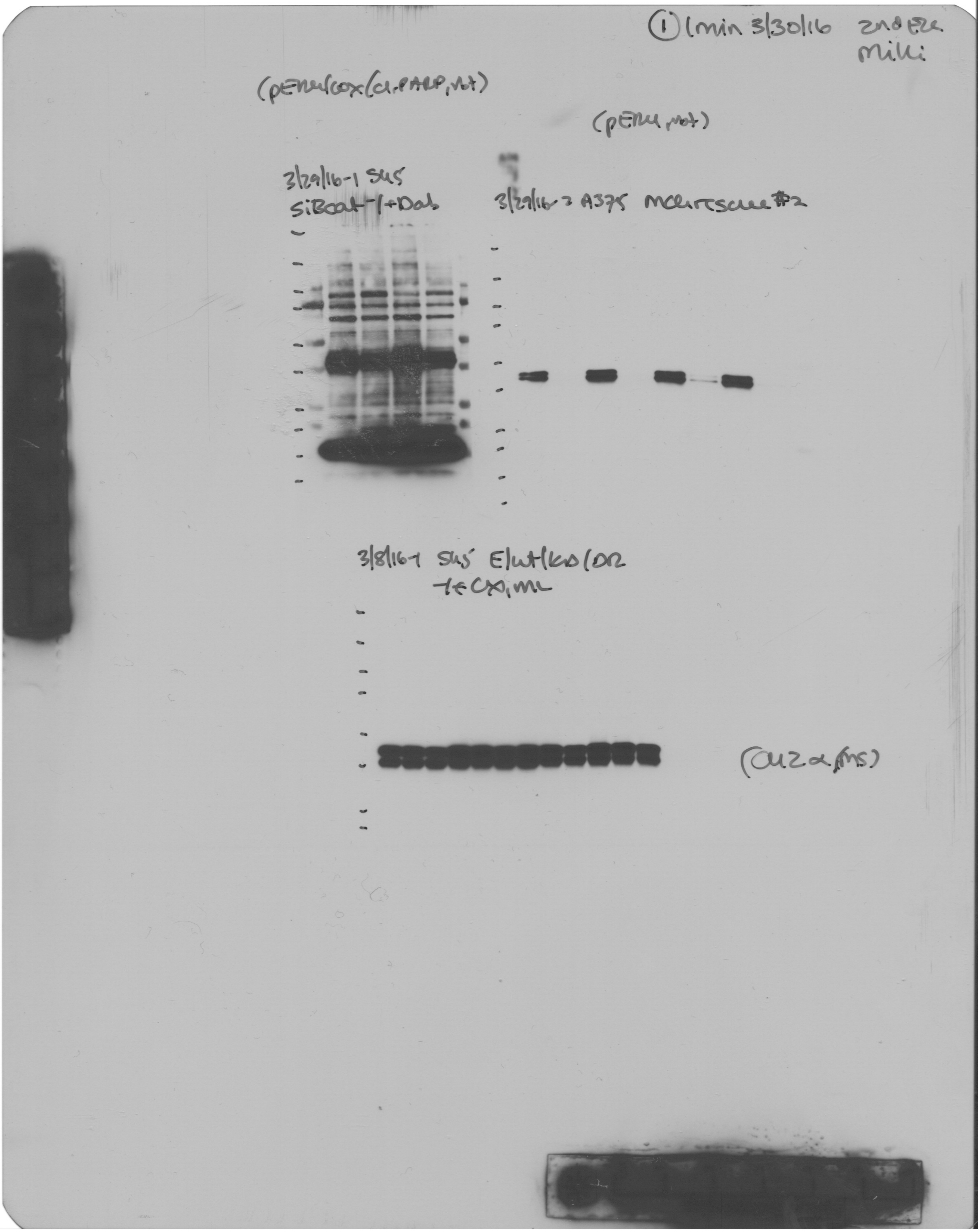

① 1min 3/30/16 2nd ECL
Milli
(pERK/cox/cl.PARP, Act)
(pERK, Act)
3/29/16-1 SH5
siBcat-1 +Dab
3/29/16-2 A375 [illegible] #2
3/8/16-7 SH5 E/wt/KD/DR
+CX, ML
(CK2α, ms)
